# Evaluating Stacked Methylation Markers for Blood-Based Multicancer Detection

**DOI:** 10.3390/cancers15194826

**Published:** 2023-10-01

**Authors:** Karen Funderburk, Sara R. Bang-Christensen, Brendan F. Miller, Hua Tan, Gennady Margolin, Hanna M. Petrykowska, Catherine Baugher, S. Katie Farney, Sara A. Grimm, Nader Jameel, David O. Holland, Naomi S. Altman, Laura Elnitski

**Affiliations:** 1 Translational and Functional Genomics Branch, National Human Genome Research Institute, National Institutes of Health, Bethesda, MD 20892, USA; 2 Integrative Bioinformatics Support Group, Biostatistics and Computational Biology Branch, National Institute of Environmental Health Sciences (NIEHS), National Institutes of Health, Research Triangle Park, Durham, NC 27709, USA; 3 Department of Statistics, Pennsylvania State University, University Park, PA 16802, USA

**Keywords:** liquid biopsy, DNA methylation, biomarker, cancer, multi-cancer, multi-cancer, bisulfite sequencing, diagnostics, screening

## Abstract

**Simple Summary:**

Tumors are known to shed DNA into the bloodstream, and since the tumor DNA is marked by aberrant methylation patterns, this can be exploited for their detection through a simple blood sample. However, specific methylation biomarkers that efficiently detect a broad range of tumors and are effective at early-stage disease are still lacking. In this study we identify two novel methylation biomarkers and combine these with an already existing biomarker to improve multi-cancer detection. We test their performances as individual and combined markers using large methylation array datasets covering multiple cancer types, mimic blood samples using data from healthy blood cell DNA, and finally test the biomarkers in cancer plasma samples. We find that the combination of markers greatly improves the ability of the test to distinguish between cancer and normal samples, and in addition we provide the research field with a complete workflow for evaluating novel methylation biomarkers based on pre-existing datasets.

**Abstract:**

The ability to detect several types of cancer using a non-invasive, blood-based test holds the potential to revolutionize oncology screening. We mined tumor methylation array data from the Cancer Genome Atlas (TCGA) covering 14 cancer types and identified two novel, broadly-occurring methylation markers at *TLX1* and *GALR1*. To evaluate their performance as a generalized blood-based screening approach, along with our previously reported methylation biomarker, *ZNF154*, we rigorously assessed each marker individually or combined. Utilizing TCGA methylation data and applying logistic regression models within each individual cancer type, we found that the three-marker combination significantly increased the average area under the ROC curve (AUC) across the 14 tumor types compared to single markers (*p* = 1.158 × 10^−10^; Friedman test). Furthermore, we simulated dilutions of tumor DNA into healthy blood cell DNA and demonstrated increased AUC of combined markers across all dilution levels. Finally, we evaluated assay performance in bisulfite sequenced DNA from patient tumors and plasma, including early-stage samples. When combining all three markers, the assay correctly identified nine out of nine lung cancer plasma samples. In patient plasma from hepatocellular carcinoma, *ZNF154* alone yielded the highest combined sensitivity and specificity values averaging 68% and 72%, whereas multiple markers could achieve higher sensitivity or specificity, but not both. Altogether, this study presents a comprehensive pipeline for the identification, testing, and validation of multi-cancer methylation biomarkers with a considerable potential for detecting a broad range of cancer types in patient blood samples.

## 1. Introduction

The negative impact of COVID-19 resulted in an estimated 10 million missed cancer screenings in the first six months of 2020 [1] and an 11% increase in diagnoses at inoperable or metastatic timepoints in the last ten months of the year [2]. COVID-19 notwithstanding, the lifetime risk of developing any type of cancer in the United States is 43% for men and 33% for women [3]. Despite this relatively high incidence, most cancer types completely lack effective tests suitable for general screening. Routine cancer screening, recommended for a few cancer types includes colon, rectal, breast, and cervical per the American Cancer Society and the US Preventive Services Task Force [4]. Additionally, lung cancer, the most prevalent cancer, affects more than 200,000 individuals annually, but the screening test has qualifiers—individuals must be aged 50 to 80 years and have a 20 pack-year smoking history [5].

The absence of generalized cancer screening combined with a lack of patient adherence to available testing contributes to the problem of a higher prevalence of tumors being detected at late stages, which in turn is known to produce poor survival rates in these patient populations [6]. In addition to these considerations, screening for most individual cancer types remains impractical because their low incidence rates coupled with low specificity tests increases the false positive to true positive ratio. Barriers to effective screening explain why cancers continue to be diagnosed at late stages of disease where treatment efficacy remains limited, medical costs increase dramatically, and five-year survival rates diminish [7].

New, non-invasive, and affordable screening tests are urgently needed that can reliably detect more cancer types, at stages early enough to provide effective treatment [8,9]. Blood-based tests, known as liquid biopsies, are emerging to fill this gap. Indeed, a simple blood sample can provide access to a plethora of analytes such as cell-free DNA (cfDNA), proteins, and circulating tumor cells (CTCs) [10]. With minimal characteristics for patient discomfort, test invasiveness, and time required, these tests could complement the current diagnostic modalities of imaging, tissue biopsy, and exploratory surgery.

The detection of markers with high sensitivity and specificity for widespread cancer screening is the current challenge in the field. Although many cancer detection tests target somatic mutations in cfDNA, with the expectation of exclusivity to tumor cells, differences in driver mutations per individual tumors reduce test sensitivity [11,12,13]. Therefore, generalized, multi-cancer screening using somatic mutations requires large biomarker panels, inclusive of hundreds to thousands of marker sites to catch multiple cancer types with rigor. These tests also suffer false positives from mutations carried in clonal hematopoietic cells, which contain known driver mutations that are not present in the solid tissue tumors [14]. In contrast to the pitfalls of somatic mutation testing, DNA hypermethylation sites can occur consistently across individual tumors originating from the same organ and in different cancer types [15,16,17,18]. Our prior work indicates that methylation-based biomarkers are more reliable for cancer detection and, as we show here, represent ideal sources for a generalized approach to detecting multiple types of cancer.

Previously, we reported a promising multi-cancer methylation biomarker, *ZNF154*, which is hypermethylated in at least 14 different cancer types in The Cancer Genome Atlas (TCGA) of tumor data [19,20]. Importantly, we demonstrated a higher incidence of *ZNF154* hypermethylation compared to panels of somatic driver mutations, underscoring the utility of a multi-cancer, DNA methylation biomarker [21]. Utilizing a PCR-based digital detection approach we demonstrated identification of late-stage ovarian cancer and early- and late-stage pancreatic cancer from 1–2 milliliters of patient plasma [21,22]. However, as some tumors were missed by the marker, we predicted that cancer detection by *ZNF154* could be improved by combination with additional multi-cancer methylation biomarkers.

In this study we mined TCGA methylation data from 14 cancer types and identified two additional methylation markers for use in combinatorial testing: *GALR1* and *TLX1*. While novel pan-cancer markers such as these continue to emerge, the need for preliminary validation methods prior to initiating extensive laboratory testing intensifies. Therefore, the objective of this study was not only to validate the three markers individually and combined, but also to explore strategies for testing the biomarkers in a more blood-related context prior to processing plasma samples. The goal of this approach is to detect a broad spectrum of tumor types. Naturally, this calls for additional, more advanced follow-up tests, enabling identification of the tumor tissue of origin, which will not be in the scope of the present study. Exploiting the large number of tumor samples contained in TCGA database, we were able to assess multi-cancer detection performance across 14 cancer types, with cross-validation and validation in four independent cancer datasets, creating a solid foundation for the identified biomarkers. In addition to simulating dilutions of TCGA data with tumor DNA concentrations decreasing from 100–1%, also with cross-validation, we tested different thresholds used in calling a positive test. We evaluated this approach in terms of the analytical performance criteria, sensitivity, and specificity. We also demonstrated the beneficial effect of combining markers on the positive predictive value (PPV). Finally, we tested the marker combinations in two cohorts of patient plasma samples using bisulfite treated cfDNA sequencing data previously generated from lung and liver cancer patient samples. We ultimately provide a complete workflow generating supportive evidence for the further implementation of these three markers into a multi-cancer detection assay.

## 2. Materials and Methods

### 2.1. The Samples, Datasets, and Preprocessing

The Cancer Genome Atlas (TCGA) data and ENCODE cell line data were collected as described in Sanchez-Vega et al. [19]. In brief, we downloaded level 3 data for 15 different cancer types from TCGA data portal (https://tcga-data.nci.nih.gov/tcga/) (accessed on 1 October 2013). The data represent the same collection of TCGA DNA methylation values processed for the discovery of *ZNF154* DNA methylation [19,20] from our lab. All pre-processing and normalization steps remain the same. For example, these data were acquired by TCGA from the Illumina Human Methylation 450 K platform and were pre-processed following TCGA level 3 standard protocols. We discarded all probes that were located on chromosomes X and Y, as well as all data points that were masked as NA (‘Not Available’). Data were frozen 1 October 2013, were BMIQ normalized [23], and known cross-reactive probes were removed [24]. Our previous analysis of batch effects across TCGA data was published in [19], as well as a large analysis in supplemental materials of [25]. Our approach of using BMIQ normalization is consistent with recent analyses of TCGA methylation data by Zhu et al., Nature Methods 2022 [26]. Thyroid cancer was excluded from the dataset since previous work did not show differentially methylated probes within this tumor type. Overall, 2711 peripheral whole blood cell samples representing male and female donors, ages 35–80 years, were from the GEO dataset GSE55763 [27]. Independent sets of Illumina Infinium human methylation array data from breast cancer samples (n = 450) and normal tissue (n = 149) were pooled together from three GEO datasets (GSE37754 [28], GSE66695 [29], and GSE69914 [30]). Illumina Infinium human methylation array data from colon cancer samples (n = 35) and normal tissue (n = 18), and lung adenocarcinoma samples (n = 9) and normal tissue (n = 11) came from GEO dataset GSE53051 [31]. Prostate cancer data came from GSE112047, with data collected from tumor (n = 31) and normal tissues (n = 16) [32]. All Illumina Infinium data were processed as described for TCGA data. Breast tumor (n = 40) and normal breast tissue DNA (n = 8) were obtained from AMSBio (Cambridge, MA, USA), as reported in Margolin et al., 2016 [20]. Each well contained five µL genomic DNA at approximately 4 ng/µL, yielding 20 ± 3 ng genomic DNA per sample (mean ± SD). Experimental datasets comprising whole genome bisulfite sequencing (WGBS) from cell free DNA of plasma were from lung cancer (n = 9) or normal plasma (non-cancer samples) (n = 4) [33,34]. The nine lung cancer plasma samples covered six cases of adenocarcinoma (stage 1A or 1B), one case of non-small cell carcinoma (stage IIIA/IV), one case of squamous cell carcinoma (stage unknown), and one case of benign fibroelastotic scar [33]. Additional WGBS data from plasma of liver cancer (n = 26 + 4) or healthy individuals (n = 32 + 4) were obtained from [34,35]. Liver cancer plasma samples covered 25 samples with BCLC stage A (early stage), one sample with BCLC stage B (intermediate stage), and four samples of unknown stage [35].

### 2.2. Probe Identification

We assessed differential methylation in all positions on the Illumina 450 K methylation array data, using data from 4050 tumors and 646 normal samples provided by TCGA in 14 cancer types. Processing 485,512 probe positions on the array in one tumor type at a time, we searched for CpG sites whose absolute differences in mean and median methylation beta values between tumors and normal samples were above 0.4, and with significant *p*-values from both *t*-test and Wilcoxon rank sum tests after Bonferroni-correction. The Wilcoxon test constraint was removed for three tumor types (pancreatic, rectal, and stomach adenocarcinoma), where no probes would otherwise have been identified. The genomic coordinate for the *GALR1* probe cg03502002 in hg19 is chr18: 74,962,134. For *TLX1*, the genomic coordinate for probe cg14861089 in hg19 is chr10: 102,895,044, and the *ZNF154* probe cg21790626 is located at chr19:58,220,494. Mean and median values are presented in Table S1. The *GALR1* locus window coordinates are chr18:74961138−74962794 and contain 25 CpGs, the *TLX1* window coordinates are chr10:102894120−102895708 and contain 9 CpGs, and the *ZNF154* window coordinates are chr19:58211993−58220837 and contain 11 CpGs (all in hg19).

### 2.3. Sensitivity, Specificity, and Sample Classification in TCGA Data

Logistic regression models were trained using methylation levels from one, two, or three markers as covariates (additive effect) for all 14 cancer types individually (coefficients for each covariate can be found in Table S2). Next, receiver operating characteristic (ROC) curves were built with the ‘pROC’ package in R and used to find optimal thresholds for classifying tumor vs. normal samples. The area under the ROC curve (AUC) was used to measure the performance of each combination of markers as a classifier. For all 14 tumor types we tested the three markers separately, in pairwise combinations, and all three markers together, which resulted in 14 AUC values for each possible combination. We performed Friedman test (tumor types set as blocks) and pairwise Wilcoxon signed-rank tests with Bonferroni corrected *p*-values to compare average AUC values from each of the possible biomarker combinations. We applied two strategies for validating test performance. First, each of the 14 TCGA cohorts (tumor and normal samples) were split randomly 50/50 five times and each time one set was used for training and setting of thresholds, while the other was used for validation. Second, we validated the test performance in independent datasets, using logistic regression coefficients for the corresponding cancer type and probe combinations from TCGA data to predict probabilities in the independent validation cohorts. This was followed by classification using the same cutoff threshold applied to TCGA discovery cohort. The sample code will be made available at https://github.com/elnitskilab/Tumor_classification_bymethylation (accessed on 10 May 2023).

### 2.4. Blood Sample Simulations

Each of the 4050 TCGA tumor samples were matched with a random normal peripheral blood sample (some blood samples were matched multiple times). Methylation data obtained from TCGA tumors were simulated into percentages of total DNA by weighting the average methylation value from a given tumor sample and normal peripheral blood cell sample at the targeted fractions. Simulated circulating tumor DNA (ctDNA) fractions ranged from 1–100%. We did not take into account the fragmentation pattern observed in cell-free DNA [36] when performing these simulations. At each concentration (100, 99.9, 99, 90, 75, 50, 25, 10, 1%), logistic regression models were trained using all 4050 tumor samples pooled into a single cohort and normal peripheral blood samples while applying 10-fold cross-validation, followed by construction of ROC curves and calculation of AUC as a measure of performance. The accuracy and kappa statistics (incl. SD) from the 10-fold cross-validation are reported in Table S3. We selected the optimal threshold based on results with 10% tumor DNA concentration and applied this common threshold to each of the 14 cancer types and reported the sensitivity for each of the markers. The coefficients for each covariate in the logistic regression models at 10% tumor DNA concentration are reported in Table S4.

### 2.5. Calculation of Positive Predictive Value

Incidence rates were extracted using the SEER*Stat software Version 8.4.0 providing access to the 1975–2019 SEER Research Plus Data (November 2021 Submission). We used the database ‘Incidence—SEER Research Limited-Field Data, 22 Registries, November 2021 Sub (2000–2019)’ and retrieved age-adjusted rates using the 2000 U.S. Std Population (19 age groups—Census P25–1130). Rates were calculated for cases diagnosed from 2010–2019 and with age at diagnosis set to 50–85+, thereby maintaining a focus at the screening-relevant part of the population. We compiled a complete list of tumor subtypes covered by TCGA cohort used for analysis, including the topography code (ICD-O-3 Site) and the morphology code (ICD-O-3 Histology). We then extracted the relevant incidence rates from SEER and summed the subtype-specific rates together to obtain the final incidence rate for each tumor type (e.g., BRCA). In the lung squamous cell carcinoma (LUSC) cohort, a single tumor sample was excluded prior to the retrieval of incidence rates, since the patient was diagnosed with ‘Adenocarcinoma, NOS’ at the ‘upper lobe, lung’, and thus in theory belonged to the lung adenocarcinoma (LUAD) type. A list of incidence rates applied for each TCGA tumor type can be found in Table S5. The positive predictive value (PPV) was calculated as:

$$\frac{sensitivity\times incidence}{sensitivity\times incidence+\left(1-specificity\right)\left(1-incidence\right)}$$

with incidence rates inserted as fractions of 1. Using the common threshold set to detect all 14 cancer types with maximum sensitivity and specificity (Youden’s index), PPV was calculated for each individual tumor type. When estimating the PPV for all tumor types combined, each tumor type-specific sensitivity was multiplied with the respective incidence rate, and the sum of this product across all 14 tumor types was used. The PPV was plotted as a boxplot showing the median with first and third quartiles, based on the PPV values obtained from the seven possible probe combinations (*GALR1*, *TLX1*, *ZNF154*, *GALR1* + *TLX1*, *GALR1* + *ZNF154*, *TLX1* + *ZNF154*, *GALR1* + *TLX1* + *ZNF154*). Alternatively, we calculated the PPV for each of the assays where thresholds were determined based on a fixed specificity at 95%.

### 2.6. PCR Assay and Sequencing

The sample cohort covered 40 breast tumors and 8 normal breast samples, each in duplicate. Our primer design assumed all non-CpG cytosines (C) to be converted with sodium bisulfite to thymine (T). The primers were annealed to regions in the genomic DNA sequence devoid of any cytosines in a CpG context. The following PCR primers were used:

 

*ZNF154*, at genomic position Chr19: 58220404-58220705 (+strand)
Forward primer: 5′-GGTTTTTATTTTAGGTTTGA-3′
Reverse primer: 5′-AAATCTATAAAAACTACATTACCTAAAATACTCTA-3′
Amplicon size (incl. primers) 302 bp (20 CpGs)

 

*TLX1*, at genomic position Chr10: 102894992-102895165 (+strand)
Forward primer: 5′-TTTTTAGTTTAGGTTTTATGGGGTAG-3′
Reverse primer: 5′-AAAACCATAACTTCCTTTATAACCC-3′
Amplicon size (incl. primers) 174 bp (13 CpGs)

 

*GALR1* at genomic position Chr18: 74961979-74962242 (+strand)
Forward primer: 5′-GGGAGTTTTTTTTGTAGGAGT-3′
Reverse primer: 5′-AAAACACTAAAATCCCCTTCC-3′
Amplicon size (incl. primers) 264 bp (27 CpGs)

*TLX1* and *GALR1* amplicons were generated and sequenced at Zymo Research in a multiplex reaction. Zymo’s PCR annealing temperature was 56 degrees, and bisulfite conversion, PCR, and sequencing were performed in the same way as *ZNF154*.

For purposes of sequencing, the primers contained the following adapters:

- Forward adapter: 5′-ACACTCTTTCCCTACACGACGCTCTTCCGATCT-3′,
- Reverse adapter: 5′-GTGACTGGAGTTCAGACGTGTGCTCTTCCGATCT-3′.

In brief, *ZNF154* amplicons were prepared from 20 ng genomic DNA, which was bisulfite converted with the EZ DNA Methylation-Direct Kit (catalog no. D5023; Zymo Research, Irvine, GA, USA).

PCR products were sequenced at the NIH Intramural Sequencing Center using the Illumina MiSeq platform with Reagent Kit Version 3 (Illumina Inc., San Diego, CA, USA) to generate paired-end, 300-bp reads. Post-run processing of data was performed using RTA version 1.18.42 and CASAVA software version 1.8.2 (Illumina Inc.). Sequencing data from PCR-generated amplicons of *ZNF154* were obtained from our previous study [20]. *TLX1* and *GALR1* analysis was carried out using standard Illumina base-calling software and methylation was analyzed using Zymo’s in-house pipeline built on Bismark version 0.7.12. 

We aligned the full-length fragments to the human genome version GRCh37/hg19 using Bismark version 0.7.12 [37]. This procedure filtered out non-aligning reads and returned the number of aligned reads together with methylation levels at each CpG in the amplicon and mean methylation across each sample. Samples with read count < 500 were excluded from the analysis of the *ZNF154* amplicon. Since *TLX1* and *GALR1* amplicons resulted in lower sequencing reads, the threshold was set proportionally lower for the analyses of these two amplicons (19 reads as cutoff). The duplicate samples were treated as separate sample cohorts in order to check for reproducibility of the results with either sample cohort. The maximum mean methylation level among the normal samples was used to set the threshold for classification of the tumor samples (resulting in 100% specificity). To combine the markers into multi-marker assay, every sample that passed the read count limit for all markers was evaluated for positivity for each of the markers and presented in the Venn diagram.

### 2.7. EpiClass Procedure to Assess Marker Performance in Plasma Samples

In accordance with the EpiClass procedure [22], we set two thresholds using training to call positive samples: (1) a minimum methylation density, MD_min_, defined from the fraction of methylated CpG sites within the region for all samples (see Table S6), and (2) a minimum epiallelic fraction, EF_min_, defined as the fraction of reads at or above the designated methylation density threshold. Given a set of training data, the EpiClass procedure solves for these two parameters simultaneously by calculating maximum sensitivity, specificity, or both for various combinations of MD_min_ and EF_min_, choosing the pair of parameter values that maximizes the desired value. The thresholds can then be applied to the test data to yield either the highest sensitivity or specificity for the cohort or the highest combination of both values. A test sample was considered positive if the threshold for the methylation density *MD_min_* was met or exceeded, and the threshold for the fraction of epialleles carrying the required level of methylation, EF_min_ was achieved. We have previously compared EpiClass and CancerDetector performance in [22], where, for the analysis of a small number of loci including *ZNF154*, we found slightly better classification using EpiClass (EpiClass mean AUC  =  0.77 versus CancerDetector mean AUC  =  0.70), although this difference was not statistically significant (*p*  =  0.059; Wilcoxon rank sum 2-sided test).

As input, we used reads from the whole genome bisulfite sequencing (WGBS) plasma samples that overlapped 500–600 bp regions, flanking the corresponding CpG probe of interest. Samples were aligned using Bismark version 0.7.12 [37] and duplicate reads were removed. For each marker and marker combination used to classify the plasma samples, we initially set EpiClass to identify the optimal cutoffs that maintained 100% specificity and maximized sensitivity. In a second approach, we used the maximum value for sensitivity + specificity. For the lung cancer cohort (n = 9) and healthy controls (n = 4), all samples were treated as a training cohort with no subsequent validation. For the liver cancer cohort (n = 30) and healthy controls (n = 36), the two cohorts were repeatedly divided into two halves containing equally sized training and testing cohorts. Thus, the results from the liver cancer cohort appear as the mean of 5 iterations ± 1 standard deviation.

## 3. Results

### 3.1. Discovery of Multi-Cancer Methylation Biomarkers in TCGA Data

**Figure 1:**
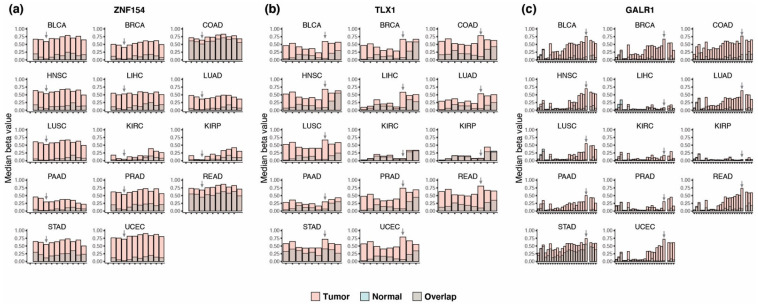
Regional hypermethylation of three multi-cancer methylation biomarkers in 14 types of cancer. Methylation beta values at (**a**) *ZNF154*, (**b**) *TLX1*, and (**c**) *GALR1* between tumor and normal control tissue samples, shown in extended windows of ≥1 kb, including the probe of interest. Each vertical bar represents the median beta value for individual CpG sites on the Illumina array, and each grid position shows a different cancer type. Bars are colored by sample type (tumors in pink and normal in cyan blue) and plotted as overlayed bars (i.e., the normal value appearing under the tumor value is shown in gray). The density of CpG sites present in these windows varies by gene: *ZNF154* = 11, *TLX1* = 9, and *GALR1* = 25. The gray arrows indicate the probes with the highest median differential methylation value for each locus, averaged across all 14 cancer types. All methylation data were obtained from The Cancer Genome Atlas.

We previously showed that *ZNF154* probe cg21790626 (hg19/chr19:58,220,494) in Illumina’s 450 K methylation array is significantly hypermethylated in 4050 TCGA tumor vs. 646 normal samples from 14 cancer types [19]. Revisiting these data, we found that the mean methylation level at Illumina probe cg21790626 (i.e., *ZNF154*) across all tumor samples was high (0.46 ± 0.004 SEM) compared to normal tissues (0.08 ± 0.005 SEM). The difference in median beta value between cancer and normal tissue within individual cancer types varied from 0.01 in kidney renal papillary cell carcinoma (KIRP) to 0.69 in uterine corpus endometrioid carcinoma (UCEC) (Figure 1a). In a ±1000-bp window surrounding the *ZNF154* probe cg21790626 site, we evaluated methylation status in 11 CpG nucleotides present on the array (Figure 1a); however, *ZNF154* at the position cg21790626, which we previously reported, showed the highest average difference in methylation beta values between tumor and normal samples across all 14 cancer types.

To identify additional markers with similar performance to *ZNF154* across multiple cancer types, we reanalyzed TCGA data from the same 14 cancer types. We required mean and median methylation differential values ≥ 0.40 between tumor and normal samples for at least one CpG position (Bonferroni corrected *p*-values < 0.05 for t-tests and Wilcoxon rank sum tests). This process revealed several CpG sites that displayed strong differential methylation signals in a wide range of tumors. We followed up on two positions as putative multi-cancer markers. At probe cg14861089 in *TLX1* (hg19/chr10:102,895,044), the difference in median beta values between tumor and normal samples across all 14 cancer types ranged from 0.03 in kidney renal clear cell carcinoma (KIRC) to 0.73 in uterine corpus endometrioid carcinoma (UCEC) (shown in Table S1). At this position, hypermethylation met or exceeded the 0.40 threshold in 9 of 14 cancer types (Figure 1b), including bladder, breast, head and neck, liver, lung, pancreas, prostate, stomach, and uterine cancer. A second methylation probe, cg03502002 in *GALR1* (hg19/chr18:74,962,134), showed an average difference in median beta value between tumor and normal of 0.43, with the smallest difference detected in KIRP (0.02) and the largest difference again found in UCEC (0.70). *GALR1* also met the 0.40 threshold in nine tumor types: bladder, breast, colon, head and neck, lung, prostate, rectal, stomach, and uterine cancer (Figure 1c). By comparison, *ZNF154*, which was not originally identified using this method, met the 0.40 threshold in only six cancer types: bladder, head and neck, liver, lung squamous cell, stomach, and uterine. These findings suggest the two new markers could have comparable or even greater potential as multi-cancer markers than *ZNF154*, however, the performance across 14 cancer types is seldom determined by a single evaluation method.

### 3.2. Methylation at TLX1, GALR1, and ZNF154 in Tumor and Normal Karyotype Cell Lines 

To expand upon cancer types in our methylation analysis in human tissues from TCGA data (Figure 1), we examined cell lines from a variety of tumor and normal cell types (Figure S2). Previously, we observed a striking pattern in which *ZNF154* was methylated in cell lines derived from tumors, but not nontumor cell lines [19]. Here, we assessed methylation data for *TLX1*, *GALR1*, and *ZNF154* in cell lines derived from non-tumor (n = 30) or tumor (n = 23) cell karyotypes [38], and in addition nine cell lines with no designation. For each marker, we determined that methylation was significant in tumor-derived cell lines vs. normal cell lines based on a cutoff median beta value > 0.02 (*p* < 0.01, Fisher’s exact test for each marker). The average beta value across all tumor-derived cell lines was very high: 0.87 for *TLX1*, 0.90 for *GALR1*, and 0.94 for *ZNF154*. Conversely, the average beta value across all non-tumor-derived cell lines was <0.1 for each marker.

Slight differences existed for each marker. *ZNF154* was unmethylated in all non-tumor-derived cell lines (range 0–0.05) and hypermethylated in all tumor-derived cell lines (range 0.47–0.99), with the exception of Nt2d1 (a teratocarcinoma cell line), which had methylation beta values < 0.1 for all three markers. The low level of methylation seen in this cell line may be related to its pluripotent nature [39]. *TLX1* showed high methylation (>0.4) in 18/23 tumor-derived cell lines and two non-tumor cell lines. The two normal cell lines were HEEpic (human esophageal epithelial cells) and NHBE (human bronchial epithelial cells). *GALR1* showed high methylation in 18/23 tumor-derived cell lines and one non-tumor-derived cell line, GM12878 (B-lymphocytes derived from peripheral blood).

Thus, *ZNF154*, *TLX1*, and *GALR1* methylation were elevated across the majority of tumor-derived cell lines. Notably, the combination of the three markers clustered the known tumor cell lines and normal cell lines into distinct groups, with only two exceptions—cell lines with no information (“Not specified”) clustered with both the tumor and non-tumor groups based on their methylation values. Notably, the tumor cell lines included cell types, which were not included in the 14 cancer types we analyzed from TCGA in Figure 1 (Figures S1 and S2), such as leukemia (CMK), neuroblastoma (BE2_C), cerebral brain tumor (PFSK-1), and glioblastoma (U87), among others.

### 3.3. Performance of the Three Biomarkers Individually

**Figure 2:**
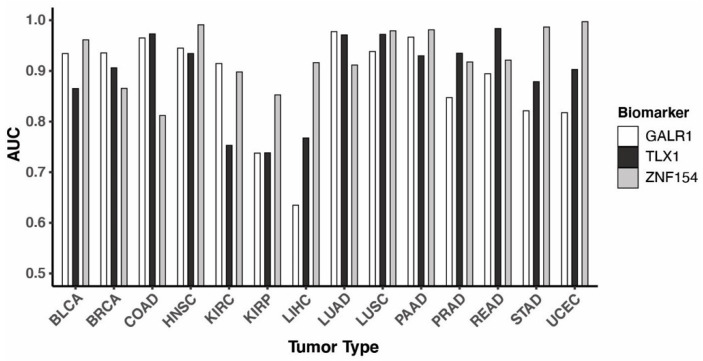
Performance of *TLX1*, *GALR1*, and *ZNF154* in classifying tumor versus normal tissue. Area under the ROC curve (AUC) values are plotted for each of the 14 tissue types (in total 4050 tumor samples, 646 normal tissue samples). Bar colors indicate the biomarker tested (white = *GALR1*, dark grey = *TLX1*, light grey = *ZNF154*). All methylation data were obtained from The Cancer Genome Atlas repository and were abbreviated as in Figure 1.

To classify tumor and normal samples from TCGA data, we trained logistic regression models using methylation beta values from the three biomarkers one tumor type at a time. Next, we determined the best probability thresholds for classification with receiver operating characteristic (ROC) analysis and used the area under the ROC curve (AUC) to measure the performance of each marker. Across the 14 tumor types, the three markers showed varying AUC values ranging from 0.63–1.0 (Figure 2). While *GALR1* and *TLX1* showed near perfect performance in classifying colon cancer (AUC of 0.97 for both), *ZNF154* only achieved an AUC of 0.81 within this tumor type. On the other hand, *ZNF154* showed optimal performance in several other cancers such as uterine corpus endometrial and head and neck cancer (Figure 2). Importantly, *ZNF154* enabled near-perfect classification of liver cancer samples with an AUC of 0.92, whereas *GALR1* and *TLX1* only produced AUC values of 0.64 and 0.77, respectively. As expected, the sensitivity and specificity at the optimal threshold of each of the three biomarkers differed across the 14 tumor types in accordance with the AUC values (Table S7). A good example of the compromise between sensitivity and specificity when settling on a threshold is the performance of *ZNF154* in colon cancer. Due to the relatively high level of methylation in normal colon tissue TCGA samples (median beta value 0.51 in TCGA samples), the threshold that allows for a high specificity (92%) comes with a compromise in sensitivity (64%). The same tendency could be observed for this marker in classification of rectal cancer samples, albeit with a better performance (100% specificity and 78% sensitivity). On the contrary, because the *GALR1* marker suffered from low levels of methylation in TCGA liver cancer tissue (median beta value 0.09), the specificity of 94% corresponds to a sensitivity of only 46%, making it a poor marker for detection of this cancer type. To summarize each marker, we found the average of the sensitivity values in tissue samples across all 14 cancer types was 78% for *GALR1*, 81% for *TLX1*, and 84% for *ZNF154*. The average specificity across all 14 tissue types was 95% for *GALR1*, 96% for *TLX1*, and 97% for *ZNF154*. Together, these data indicate that no marker has ideal performance in all cancer types and supports the use of multiple markers to complement each other’s performance. While the vast majority of cell-free DNA (cfDNA) in plasma from healthy individuals derives from hematopoietic cells [36], certain pathological states (related or unrelated to cancer) can alter the cell type contribution to cfDNA [40]. Thus, we find it noteworthy to ensure differential methylation levels between tumor and normal tissue, as demonstrated for these three markers.

### 3.4. Combining Methylation Biomarkers into Multi-Marker Assays

**Figure 3:**
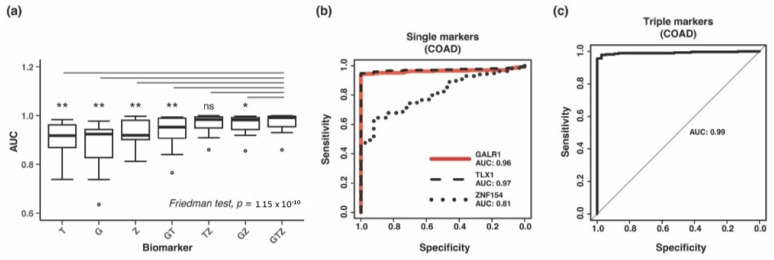
Performance of *TLX1*, *GALR1*, and *ZNF154* combinations in tumor classification. (**a**) Box plots are shown using the area under the receiver operating characteristic (ROC) curve (AUC) for each possible marker combination (G, *GALR1*; T, *TLX1*; Z, *ZNF154*). AUC values were calculated for each of the 14 cancer types (4050 tumor samples, 646 normal tissue samples), and thus each boxplot contains 14 AUC values. All marker combinations were compared by the Friedman test followed by a pairwise comparison between the triple marker assay and each of the other combinations using Wilcoxon signed-rank test with Bonferroni correction. * *p*-value < 0.05, ** *p*-value < 0.01, ns ‘not significant’. (**b**,**c**) ROC curves showing classification performance in colon cancer vs. normal tissue by either the three markers separately (**b**) or combined (**c**). All methylation data were obtained from The Cancer Genome Atlas.

We next evaluated the combination of two or three markers. To combine the markers, we included them as separate covariates in the logistic regression model (coefficients for each covariate can be found in Table S2). Again, we applied a consistent thresholding approach to assess performance of biomarker combinations utilizing ROC curves and AUC within each cancer type. When compared to the performance of individual markers, the three-marker combination significantly increased the discrimination of tumor and normal samples, as evaluated by the AUC values calculated for each cancer type (Figure 3a, *p*-value = 0.0026; pairwise Wilcoxon signed-rank tests with Bonferroni correction). Compared to *ZNF154* alone, the addition of *TLX1* and *GALR1* into a three-marker assay improved the AUC value by more than 0.07 in 4 cancer types (Table S7). For example, breast cancer increased from 0.87 to 0.98 and colon cancer increased from 0.81 to 0.99 (Figure 3b,c and Table S7). The triple-marker assay even reached a perfect AUC value of 1.0 for the classification of rectal and stomach cancer (Table 1). Pairwise marker combinations including *GALR1* showed significantly lower AUC values compared to the triple marker assay, while the combination of *ZNF154* and *TLX1* showed similar performance to the triple marker assay, suggesting that combining these two markers might be sufficient (Figure 3a).

Based on each of the ROC curves, we picked a threshold that would maximize both sensitivity and specificity and tested all samples. The three-marker combination displayed a very high sensitivity across all cancers examined (average = 93%) (Table 1). The sensitivity was highest in rectal and stomach cancers (100%), as well as uterine cancer and lung squamous cell carcinoma (99%). Furthermore, in eight out of fourteen cancer types the sensitivity was ≥97%, including colon cancer. The lowest sensitivity was 74% in kidney renal papillary tumors (KIRP) and 82% in kidney renal clear cell tumors (KIRC). Compared to classification by *ZNF154* alone, the sensitivity of detection of the 14 cancer types improved with the three-marker combination, except uterine and prostate, which stayed the same (99% and 90%, respectively) (Table S7). Importantly, this high level of sensitivity was achieved without negatively affecting the specificity of the triple marker assay, which across all 14 normal tissues averaged 97%. In individual cancer types, specificity ranged from 84% in kidney renal papillary tissue to 100% in 7/14 healthy tissue types, including lung and colon tissue (Table 1). In summary, the three-marker combination demonstrated a strong ability to correctly identify tumor samples and normal samples, while missing few of either type.

To assess performance of our model within TCGA dataset, we split each tumor and normal cohort into two equally sized cohorts in a random manner and used only half of the samples for training and the other half for performance testing. We repeated the randomization split over five iterations, and the performance in both training and validation cohorts is reported in Tables S8 and S9 as mean and standard deviation. While sensitivities remained fairly stable (ranging from 72.3% in KIRP to >95.0% in eight cancer types), the specificities were somewhat reduced in the validation cohorts for some of the tumor types, including bladder, breast, lung, and prostate cancer (ranging from 80.0 to 88.8%). The test performance in the training cohorts across the five iterations showed little to no variation, which can be seen from the resulting ROC curves and AUC values, which remain consistently high (ranging from 0.87 to 1.0), and the fact that all AUC standard deviations were <0.02 (Table S8 and Figure S3).

### 3.5. Validation of Three-Marker Combination in Independent Tumor Datasets

**Figure 4:**
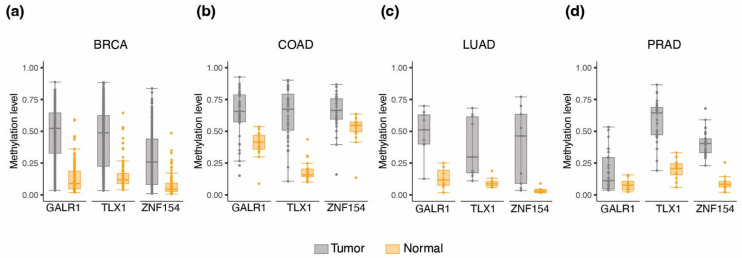
Hypermethylation at the three methylation probes in independent validation cohorts. Box plots of methylation beta values in (**a**) BRCA (GSE37754, GSE66695, GSE69914), (**b**) COAD (GSE53051), (**c**) LUAD (GSE53051), and (**d**) PRAD (GSE112047) for each methylation probe. Tumor types are abbreviated as in Figure 1. The number of samples in each group is listed in Table 2.

All analyses up to this point were based on the same TCGA datasets. Therefore, we validated the performance of the *GALR1*, *TLX1*, and *ZNF154* markers in independent sets of Illumina 450 K methylation array data available for four cancer types: breast cancer (450 tumors, 149 normal samples), colon cancer (35 tumors, 18 normal samples), lung adenocarcinoma (9 tumors, 11 normal samples), and prostate adenocarcinoma (31 tumors, 16 normal samples). In all of these datasets, the average methylation value was elevated in tumor samples compared to normal tissue samples for all three probes (Figure 4a–d), and in general the methylation levels were comparable to those found in the corresponding TCGA cohort (Figure S4). To test assay performance in these validation cohorts, we utilized TCGA-based trained regression models and calculated thresholds described in the section above. The classification performance for the three-marker combination can be viewed in Table 2. The sensitivity and specificity in this breast cancer validation set were 80% and 94%, respectively, which was slightly reduced from 91% and 96% in TCGA dataset. For the colon cancer validation set the sensitivity and specificity were 94% and 83%, respectively, which was also slightly reduced from 97% and 100% in TCGA dataset. The lung adenocarcinoma validation data, albeit being a very small cohort, showed a shift in the balance between sensitivity and specificity, with sensitivity going up from 97% to 100%, while specificity dropped from 100% to 73%. Prostate cancer (PRAD) showed identical sensitivity while specificity improved to 100%. In summary, all validation sets achieved comparable sensitivity and specificity using the thresholds determined from TCGA data, although at slightly lower values than obtained with the training data. Results from the other biomarker combinations (single and pairwise) with the validation cohorts are shown in Table S10. No information was available to match the type, grade, or stage of tumors between the training and the testing sets. From these analyses we consider the performance of the methylation thresholds robust for further evaluation in cancer detection.

### 3.6. Methylation at TLX1, GALR1, and ZNF154 in Early-Stage Tumors

**Figure 5:**
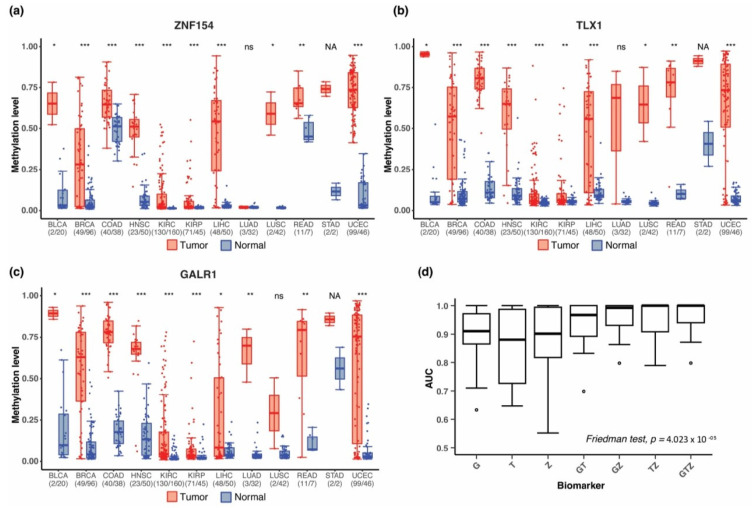
Hypermethylation at the three methylation probes in early-stage tumors. Box plots of methylation beta values at (**a**) *ZNF154*, (**b**) *TLX1*, and (**c**) *GALR1* for each cancer type in stage I tumors and normal tissue samples. The number of tumor/normal samples is listed in parenthesis below each cancer type. * *p*-value < 0.05, ** *p*-value < 0.01, *** *p*-value < 0.001, ns ‘not significant’ (Wilcoxon rank sum test with continuity correction). NA indicates insufficient sample numbers for statistical testing. Tumor types are abbreviated as in Figure 1. (**d**) Area under the receiver operating characteristic curve (AUC) calculations for stage I tumors using each possible marker combination (G, *GALR1*; T, *TLX1*; Z, *ZNF154*). Each boxplot contains 11 AUC values (STAD was excluded). Groups were compared using the Friedman test. All methylation data were obtained from The Cancer Genome Atlas.

Detecting cancer at an early stage provides the most promise for improving patient survival. After identifying our three biomarkers, we investigated if they showed differential methylation at early-stage tumors by analyzing only stage I tumors in TCGA dataset. This was not considered a discovery but a deeper assessment conducted in the same dataset, as used for Section 3.3 and Section 3.4. Stage I samples were available for 12 of 14 cancer types (unavailable in pancreas and prostate), resulting in a total of 480 tumor samples and 588 normal samples. *GALR1*, *TLX1*, and *ZNF154* were significantly hypermethylated in stage I samples for 10 of 12 tumor types (*p*-values ≤0.05 Wilcoxon rank sum test with continuity correction) (Figure 5a–c). Additionally, *GALR1* showed significant differential methylation in lung adenocarcinoma samples, despite the low number of tumor samples (n = 3). With only two stage I tumors in the stomach cancer cohort and two normal samples, the sample size was too small to be tested statistically. Again, the three markers displayed complementary strengths. *GALR1* and *TLX1* provided higher discrimination (Figure 5b,c) of normal colon and rectal tissue samples that *ZNF154* may lack (Figure 5a). The difference in median beta value between tumor and normal samples for colon was 0.13 for *ZNF154*, while *TLX1* and *GALR1* showed a difference of 0.70 and 0.60, respectively. By contrast, *ZNF154* displayed the largest amount of differential methylation for stomach cancer (difference in median methylation 0.63), improving performance over *TLX1* or *GALR1* (difference in median beta value at 0.50 and 0.30, respectively). We also tested the combination of the three markers by logistic regression in stage I tumors for 11 of 14 cancer types with available data (stomach adenocarcinoma samples were excluded due to the small sample size). For individual markers, the median AUCs for classification of stage I tumors were 0.91 (*GALR1*), 0.88 (*TLX1*), and 0.90 (*ZNF154*) vs. 1.00 using all three markers together (Figure 5d). We applied the Friedman test to compare all marker combinations using tumor types as blocks and found a significant difference between the marker combinations (*p*-value = 4.023 × 10^−5^). Although pairwise comparison using Wilcoxon signed-rank test with multiple comparison correction showed no significant difference between any of the markers, a trend towards reduced variability in AUC could be observed when combining more markers (Figure 5d). Our analyses provisionally demonstrate that each of the three markers are capable of detecting early-stage tumors in 10 out of 12 cancer types, and although they may not be the highest performing early stage markers, the finding that each biomarker has distinct strengths regarding detecting early-stage tumors further supports the use of these three markers in combination with detecting a wide range of patient tumors.

### 3.7. Testing Multi-Cancer Assay Performance in Simulated Blood Samples

**Figure 6:**
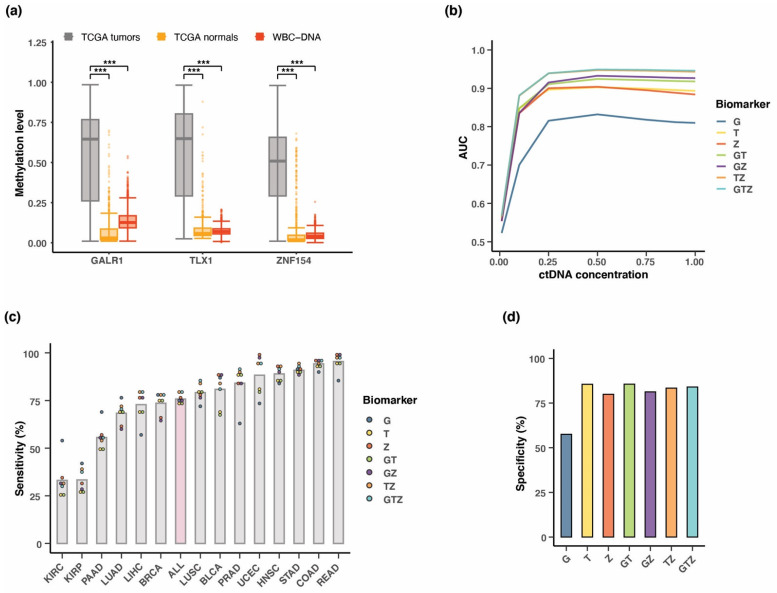
Simulated dilutions of tumor DNA into WBC DNA. (**a**) Box plots of methylation levels (beta value) at *GALR1*, *TLX1*, and *ZNF154* in DNA from tumor (n = 4050, all tumor samples combined into one cohort), normal tissue (n = 646, all normal samples combined into one cohort), or peripheral white blood cell samples (WBC-DNA) (n = 2711). Methylation data were obtained from The Cancer Genome Atlas or GEO. *** *p*-value < 0.0001 (for Wilcoxon rank sum test). (**b**) AUC values displayed by biomarker combination across a range of simulated dilutions (100–1%) of tumor DNA into WBC DNA. AUC values were derived from ROC curve analysis of all tumor samples (n = 4050) vs. peripheral white blood cell samples (n = 2711). (**c**) Sensitivity of detecting tumor samples with a ctDNA concentration of 10%. Each bar represents a tumor type (grey bars). The pink bar shows performance when considering all tumor samples in one pool. Bar height shows the mean sensitivity (%) and dots represent the performance of individual marker combinations. (**d**) Specificity in classification of peripheral white blood cell samples. Each bar represents an individual assay. For (**b**–**d**), markers are abbreviated as G = *GALR1*, T = *TLX1*, and Z = *ZNF154*.

Until this point, we assessed the ability of the three markers to distinguish between tumor and corresponding normal tissue. However, for a blood-based test, an important consideration is the differential signal between tumor DNA and healthy white blood cell DNA, since lymphoblasts provide >90% of healthy cell free DNA (cfDNA) in plasma [41]. To characterize methylation at our three candidate markers in white blood cells, we analyzed Illumina methylation array data from 2711 peripheral whole blood cell samples gathered from male and female donors, ages 35–80 years (GSE55763) [27] (Figure 6a). For all three markers, median methylation was significantly higher in TCGA tumor samples (n = 4050, all tumor samples combined in one cohort) than normal tissue samples (n = 646) or normal white blood cells (n = 2711) (*p*-values < 0.0001; Wilcoxon rank sum test). *GALR1* methylation level in white blood cells was slightly elevated compared to *TLX1* and *ZNF154* (Figure 6a). Using the peripheral whole blood cell DNA as a proxy for cfDNA, this analysis confirms that methylation at these markers in tumor cell DNA is distinct from methylation in the healthy cfDNA that would be sampled in blood-based testing.

Next, we sought to mimic patient plasma samples by simulating dilutions of the tumor DNA into a background of normal WBC DNA. Previously, we applied this approach using tumor and normal tissue DNA methylation signal at the *ZNF154* locus to predict the suitability of this marker for liquid biopsy analysis [20]. Here, we instead used WBC DNA as the normal background. In this way, the tumor DNA would now represent the circulating tumor DNA (ctDNA) while the WBC DNA mimicked the normal cfDNA. TCGA methylation data were simulated into percentages of total ctDNA by weighting the methylation beta value from tumor samples as a fraction of the methylation beta value of peripheral blood cells at the same CpG site. Dilutions decreased from 100–1% tumor DNA. Since our main aim of this study was to develop a potential multi-cancer testing approach, we combined all tumor samples into one group and assessed the ability of each marker or combination of markers to classify every tumor sample apart from normal blood samples. At each concentration we performed a new ROC analysis and obtained the AUC value for each marker or combination of markers (Figure 6b and Table S11). This analysis included a 10-fold cross-validation from which accuracy and kappa statistics (incl. SD) are reported in Table S3. At ctDNA concentrations down to 25% the AUC values for all the marker combinations remained stable. However, performance of the assays dropped markedly in the span of simulated 1–10% ctDNA, clearly illustrating the challenge of detecting ctDNA in dilute samples such as plasma. Interestingly, these data also revealed a worse performance of *GALR1* compared to the other two markers, which is likely explained by the elevated methylation level at this locus in WBC DNA (Figure 6a).

We considered head-to-head and combined marker comparisons at the 10% ctDNA concentration, where *ZNF154* and *TLX1* each reached an AUC value of 0.84 and 0.85, respectively, while *GALR1* only reached an AUC of 0.70 (Figure 6b). Importantly, the effect of combining the markers remained positive in terms of classification performance with the triple marker assay as well as *ZNF154* and *TLX1* combined in a two-marker assay both obtaining an AUC value of 0.88 at 10% ctDNA concentration. The coefficient values for each covariate in the logistic regression model at 10% ctDNA concentration can be found in Table S4. Based on the ROC curves for each of the assays we settled on thresholds that would lead to maximum sensitivity and specificity in detecting tumor DNA at 10% concentration. After classifying each sample as tumor or normal using our multi-cancer model, we then looked at performance in terms of sensitivity within each of the 14 tumor types. As shown in Figure 6c, the mean sensitivity greatly varied depending on the tumor site. Seven of the fourteen cancer types had a mean sensitivity of >80%, including gastrointestinal cancers such as colon, rectal, and stomach cancer. On the other end of the scale, the assays had lower sensitivities in kidney carcinomas and pancreatic adenocarcinoma with an average of 33% and 56% correct classification of tumor samples, respectively (Figure 6c and Table S11). Considering all 14 tumors combined, the marker combinations were performed with 76% average detection sensitivity. The assay with the highest correct classification of tumor samples was *TLX1* combined with *ZNF154* and the triple marker assay, both of which reached 79% sensitivity across all tumor samples. Values for specificity ranged from 80–86% between alternate marker combinations except *GALR1* alone, which had a specificity among the normal blood cell samples of 57% (Figure 6d). Interestingly, this trade-off in specificity was followed by a considerably higher sensitivity towards the detection of kidney renal clear cell carcinoma and pancreatic adenocarcinoma (53% and 69%, respectively) compared to the alternate marker combinations (Figure 6c). We also investigated the performance of the markers at 1% ctDNA concentration (Figure S5). However, at this concentration, the overlap between tumor and normal blood sample methylation signal from single probe methylation array data became so extensive that the maximum AUC value obtained was 0.57 (*TLX1* combined with *ZNF154* and the triple marker assay) (Table S11). We did not find this assay system sufficiently sensitive to measure ctDNA at levels < 1%.

### 3.8. Positive Predictive Value of the Three-Marker Combination

**Figure 7:**
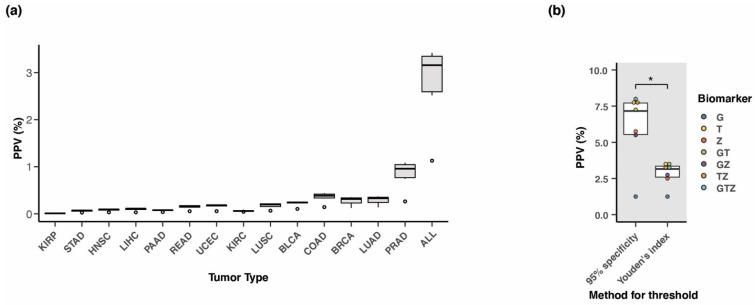
Estimating the positive predictive value when detecting multiple cancers in a single assay. (**a**) Boxplots of estimated positive predictive value (PPV) (%) of the seven different marker combinations for each tumor type individually or combined (ALL). PPV was calculated using incidence rates. (**b**) Boxplots of PPV (%) for detecting all 14 tumor types combined. Two different methods for choosing the classification threshold were compared; a threshold that results in 95% specificity for all assays (“95% specificity”) or a threshold that maximizes both sensitivity and specificity (“Youden’s index”). The resulting PPV value for each of the seven marker combinations was compared between the two types of thresholding using Wilcoxon signed-rank test. * *p*-value < 0.05. PPV for each individual assay is shown as color-coded dots. Markers are abbreviated as G = *GALR1*, T = *TLX1*, Z = *ZNF154*.

The positive predictive value (PPV) is the probability that a positive test result indicates the presence of a tumor. Additionally, the PPV provides an indication of the potential for false positive tests, and thus reveals the amount of people that would have to continue through diagnostic follow-up procedures only to confirm the absence of a tumor. The PPV of a screening test can be measured from large-scale population studies enrolling non-symptomatic individuals for screening. However, PPV can also be estimated from the analytical performance parameters, sensitivity, and specificity, calculated from case-control studies. In that case, the frequency of disease within a given population must be considered. The estimated PPV can be calculated using the following formula:

$$PPV=\frac{Sensitivity\times f}{Sensitivity\times f+\left(1-Specificity\right)\times (1-f)}$$

where f is the frequency of the disease within the investigated population. The frequency of individuals suffering from cancer is most often represented by the prevalence. However, in the case of a consistently screened population, we expect the performance of our test to be dominated by new incidences rather than already existing and diagnosed cases of cancer. Thus, we calculated the PPV for each of the seven alternate marker combination assays using age-adjusted incidence rates extracted from the Surveillance, Epidemiology, and End Results Program (SEER) database (Table S5). We applied the previously reported sensitivities and specificities for the simulated blood samples consisting of 10% ctDNA (Figure 6c,d, Table S11). While the specificity of each assay remained consistent across all tumor types, the sensitivities varied, as shown in Figure 6c. In addition, the incidence of each tumor type differed, which also had an impact on the computed PPV (Figure 7a). Thus, compared to the cancer types with low incidence rates, we observed improved PPV in the more common cancer types such as colon, breast, lung (LUAD), and prostate cancer, where the median PPV for all seven alternate marker combinations was 0.4%, 0.3%, 0.3%, and 1.0%, respectively. Most importantly, there was a clear increase in median PPV to 3.2% when considering all 14 tumor types together in a single multi-cancer screening setup, mainly due to the aggregated incidence rate (Figure 7a). Until this point, the estimated PPV values were all calculated with sensitivities and specificities derived from setting a threshold that would maximize the Youden’s index (sensitivity + specificity). However, the calculation of PPV is more sensitive to changes in specificity rather than sensitivity. Therefore, we recalculated the PPV for the multi-cancer assay aiming to detect all 14 cancer types while applying a strict threshold requiring 95% specificity for each of the alternate marker combinations. The computed PPV values for each of the 7 alternative marker combinations showed a considerable increase when using the 95% specificity threshold compared to the threshold set by the Youden’s index (Figure 7b). More specifically, the median PPV increased from 3.2% to 7.2% when specificity was forced to be 95% (*p* = 0.016; pairwise Wilcoxon signed-rank test). The best performing assay under these conditions was the triple marker assay (PPV at 8.0%), followed by the *TLX1* and *ZNF154* combined assay (PPV at 7.7%), and *TLX1* alone (PPV at 7.8%). Naturally, and as a consequence of fixing the specificity at 95%, the sensitivities dropped for all of the marker combinations. Sensitivity ranged from 42–61%, except for *GALR1*, which obtained a sensitivity of only 10% (Figure S6).

### 3.9. Performance of Three-Marker Combination in Cancer Tissue

**Figure 8:**
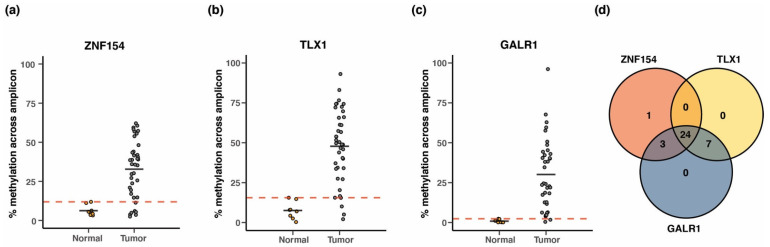
Breast tumor identification using the three biomarkers for bisulfite amplicon sequencing. Average CpG methylation level across amplicons generated by PCR amplification for (**a**) *ZNF154* (38 tumor, eight normal samples), (**b**) *TLX1* (40 tumor, eight normal samples), and (**c**) *GALR1* (37 tumor and seven normal samples). Each dot represents a tissue sample, and the methylation level is shown as a percentage. Solid grey lines represent the mean. The red dashed lines indicate the thresholds for classification of tumor samples (max methylation level in normal tissue). (**d**) Venn diagram showing the number of tumor samples correctly classified by each of the three methylation markers. Only tumor samples with read counts above QC level for all three markers were included in this graph (n = 35).

In the context of a clinical liquid biopsy, the percentage of ctDNA present is often below 1% [42]. As we have stated, we do not believe the array data are sensitive below 1%. Under such circumstances a PCR-based assay might be more suitable than an array-based assay, targeting the methylation status of single CpGs for tumor classification. Using PCR, methylation can be measured across an entire CpG island, where additional CpG positions may be present that are not represented on the Illumina microarray. These correlated positions can increase tumor discrimination [20] and enable thresholds based on methylation density, which we demonstrated in [21,22]. Thus, we next compared results from an amplicon-based methylation assay followed by sequencing to combine results from the three-markers *TLX1* (containing 13 CpGs), *GALR1* (containing 27 CpGs), and *ZNF154* (containing 20 CpGs). We tested the assay on bisulfite-converted DNA extracted from an independent collection of 40 breast tumors and eight normal breast tissue samples, as used in Margolin et al. [20]. For each marker, we averaged the methylation signal across the CpG sites within the amplified region, setting the threshold for identifying tumor samples above the maximum methylation level detected among the healthy controls (ensuring 100% specificity). Considering each of the markers individually, *ZNF154* reached 82% sensitivity, while *TLX1* and *GALR1* correctly classified 90% and 95% of the tumor samples, respectively (Figure 8a–c). The relatively high sensitivity for *GALR1* was likely caused by the lower methylation level detected in the normal tissue samples enabling a lower threshold setting. We then combined the markers such that if one marker showed methylation value above threshold, the sample would be regarded as positive. For this analysis, we only considered the 35 tumor samples that passed the QC criteria in terms of read count (see Material and Methods section) across all three markers. Interestingly, the combination of *ZNF154* with either *TLX1* or *GALR1* resulted in the successful detection of all tumor samples (100% sensitivity) (Figure 8d). To test this approach further we performed the same analysis on a second batch of biological replicates from the same cohort (Figure S7). This time, only the combination of *ZNF154* and *TLX1* was able to achieve 100% sensitivity. We concluded that two markers may be sufficient, but larger sample sizes and plasma-based samples may still benefit from the addition of a third amplicon. Thus, we demonstrated the markers are in positions amenable to PCR amplification after bisulfite conversion and were successfully implemented in a PCR-based assay. To investigate whether the inclusion of multiple CpGs surrounding the original probe of interest increased performance, we re-analyzed these data, looking only at sequencing results for the single CpGs previously assessed in TCGA methylation array dataset. Again, the threshold for counting samples as “positive” was set to the maximum beta value obtained among the normal tissue samples. Indeed, all three markers performed with increased sensitivity when utilizing information on all CpGs in the amplicon region compared to assessing only the beta value at the designated CpG position (Figure S8). We concluded that analyses of single CpG positions or low coverage WGBS might eventually yield to targeted amplicon-based analyses such as [21,22].

### 3.10. Testing of the Three Biomarkers in Patient Plasma Using Whole Genome Bisulfite Sequencing

**Figure 9:**
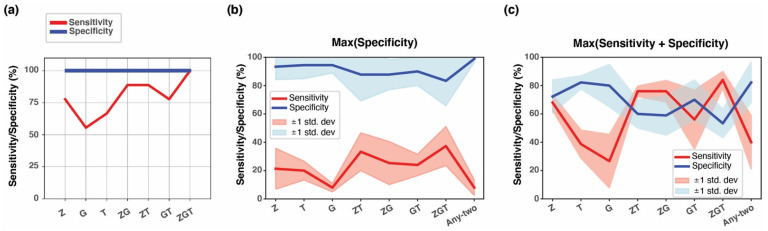
Analysis of lung and hepatocellular carcinoma whole genome bisulfite sequence data from patient plasma. (**a**) Lung cancer (n = 9) and healthy control (n = 4) plasma samples were assessed for ctDNA detection. Specificity was maintained at 100% for all markers, while sensitivity varied. Plotted are sensitivity (red line) and specificity (blue line). (**b**) Liver cancer (n = 30) and healthy control (n = 36) plasma samples were assessed for ctDNA detection. Each cohort was randomly divided into two halves (training vs. testing) over 5 iterations. Thresholds that maintained specificity at the highest detectable level (specificity) were chosen and the resulting sensitivity (red line) and specificity (blue line) were plotted. Pink or light blue areas represent ±1 SD. (**c**) Same as in (**b**) but with the threshold criteria that satisfied maximum (sensitivity + specificity) values. Markers are abbreviated as T = *TLX1*, G = *GALR1*, and Z= *ZNF154*.

To test if the three methylation biomarkers could detect the presence of a tumor from a liquid biopsy, we obtained whole genome bisulfite sequencing (WGBS) data from plasma samples belonging to lung cancer (n = 9), and hepatocellular carcinoma (n = 30) patients, along with healthy controls (n = 4, n = 36, respectively) [33,34,35]. Note that these datasets have previously been published as having low-coverage (4–10X). Here we calculated read coverage data for each of the three biomarker regions ± 300 bp, which was also in the range of 4–8X (Table S6). As seen for the PCR amplicon data, WGBS data allow for analysis of a stretch of DNA. For each target region (*TLX1*, *GALR1*, *ZNF154*) we assessed reads overlapping 600 bp regions flanking either side of the CpG sites of interest (see Table S6 for genomic coordinates and methylation density thresholds). We previously demonstrated that the extremely dilute nature of methylated ctDNA in plasma requires special analysis methods for detection, which led us to develop a novel classifier method called EpiClass [22]. This approach is more sensitive than simply setting a cutoff based on the average methylation level per sample. In brief, for each marker EpiClass will give two thresholds based on ROC curves: one for the density of methylation across the locus (i.e., percent methylation) and one for the fraction of sequence reads exceeding the percent methylation threshold (i.e., epiallelic fraction). These values can be selected based on the user’s desire to maximize sensitivity, specificity, or both. The convergence of these two thresholds on desired sensitivity or specificity values can be seen in the example heatmap (Figure S9). For each target region (*TLX1*, *GALR1*, *ZNF154*) we used EpiClass to determine the methylation density threshold (reported in Table S6). Initially, we sought to establish a threshold for the epiallelic fraction that maintained 100% specificity while maximizing sensitivity. With these thresholds, the *ZNF154* locus displayed the best performance of any single marker in lung cancer plasma with an AUC of 0.78 enabling a sensitivity of 77.8% (Figure 9a). *TLX1* had a slightly lower AUC of 0.72, whereas using *GALR1* alone to classify the samples only reached an AUC of 0.56. When combining the markers, we considered a sample to be positive if at least one of the individual markers showed methylation values above the selected thresholds. The three-marker combination, but no other, achieved 100% correct classification in this dataset (Table 3). While these results demonstrated the unique complementarity between the three markers, the small sample size and lack of a test set created the possibility of overfitting of the data, necessitating further assessment.

To further investigate the performance of the three-marker panel in a larger dataset, we tested data from the WGBS hepatocellular carcinoma plasma (n = 30) and healthy controls (n = 36). Because the sample size was still too small to divide into actual training and testing sets, we split the data and performed 5-fold cross-validation, randomly assigning half of the cases and controls into two groups (one for training and one for testing) and repeated this sampling in five iterations. We applied the EpiClass approach to each training set, seeking a threshold for the epiallelic fraction that would maximize specificity. We applied these thresholds to the classification of the five testing sets. The resulting sensitivities and specificities for the test sets are plotted as a mean of the five iterations ± 1 standard deviation (Figure 9). AUC values in the triple marker assay for each fold of classification are demonstrated with a support vector machine (Figure S10). In this dataset, our demand for high specificity resulted in a compromise in sensitivity which ranged from 8–37% (Figure 9b and Table 3). The maximum specificity was obtained when using the markers individually (range 93–94%). Maximum sensitivity was obtained when combining *ZNF154* and *TLX1* (33%) or all three markers (37%), albeit at a lower specificity (88% and 83%, respectively). In the attempt to include more than one marker without compromising specificity, we set up a different test criterion called “any-two”. Here we considered all three markers, and if two of them exceeded their respective threshold, the sample would be regarded as positive. Applying the any-two criterion increased the stringency of the test, achieving a maximum specificity of 99%, but suffering from an 8% sensitivity (Figure 9b). To accommodate an improved sensitivity, we re-analyzed the dataset with the EpiClass approach, seeking to maximize both sensitivity and specificity for each marker. We found that among the single markers, *ZNF154* yielded the highest combined sensitivity and specificity values, averaging 68% and 72%, respectively (Figure 9c). *GALR1* and *TLX1* each showed poor sensitivity with mean values of 27% and 38%, respectively, but maintained increased specificity at 80% and 82%. The addition of either *TLX1* or *GALR1* with *ZNF154* improved its sensitivity to 76% but decreased specificity to 59–60%. The three-marker panel further improved sensitivity to 84%, with a slight additional cost to specificity, which decreased to 53%. Notably, applying the any-two criterion resulted in an increased specificity compared to *ZNF154* alone (82%), while suffering a decrease in sensitivity which only reached 40%. These results indicate that plasma testing is feasible for hepatocellular carcinoma detection. Because 25 of the 30 samples represented early-stage tumors in this WGBS plasma dataset, we concluded that the results demonstrate potential for use in patient testing. Notably, a variety of combinations of these multi-cancer markers can yield similar overall classification results, although individual thresholds for sensitivity and specificity based on each marker can be selected to meet the user’s preference to exclude false positives.

## 4. Discussion

The lack of effective generalized screening tests for most types of cancers means that detection is routinely delayed until the appearance of symptoms, which typically occurs at a late stage of disease with low therapeutic efficacy. Even then, a complex set of tests may be needed to determine the origin of symptoms. An emerging technology to detect more cancers before symptoms appear is through blood-based detection of circulating tumor DNA (ctDNA) [10]. The most comparable indicators in current use are tumor-associated antigens, including PSA, CA-125, and CA-19, which can be elevated in noncancer conditions and thus lack the sensitivity and specificity of the emerging DNA-based tests [43]. We previously showed that *ZNF154* methylation detection in ovarian cancer plasma outperformed patient CA-125 measurement (69.6% vs. 47.8%, correct classification, respectively), and combined, the two markers showed improvement over *ZNF154* alone (87.0%, correct classification) [22]. Currently, a handful of liquid biopsy platforms are FDA approved as companion diagnostic tests to evaluate eligibility of patients for certain treatments [44,45,46]. Furthermore, a DNA methylation-based sequencing platform has emerged as a pan-cancer commercial product though not yet approved for clinical screening applications [47]. In addition, studies have demonstrated detection of early-stage tumors using cfDNA and protein-based liquid biopsies [48,49]. While these encouraging results demonstrate the potential for early disease detection and a workflow that enables high throughput, cost-effective analyses with a broad multi-cancer perspective are still crucial for applicability in laboratory and clinical settings. In this study, we present a simplified approach that requires no more than three DNA methylation markers which holds the potential to detect many types of cancer in a single blood-based assay. We identify two novel, multi-cancer markers in the intronic or promoter regions of *TLX1* and *GALR1*, respectively, and report the benefit of combining them with *ZNF154* as a multi-cancer assay. In addition to the potential as diagnostic markers, the hypermethylation at these locations is consistent with previously reported roles of *TLX1*, *GALR1*, and *ZNF154* in multiple cancers [50,51,52]. Furthermore, *ZNF154* was originally reported as a methylation marker specific for bladder cancer [53], later showing relevance in many cancer types [19,20].

Given the large number of DNA methylation markers that have been published [54], the likelihood that additional targets are yet to be discovered remains high. The development of DNA methylation markers for analysis of ctDNA follows a series of steps, detailed by Lissa and Robles [55], which are further developed in recent recommendations for standardization of ctDNA testing by next generation sequencing [56]. However, both of these guidelines are based on cases where the markers have already been identified and validated in preliminary studies. Considering the scarce availability of samples for preclinical and clinical analysis, alternative methods for pre-screening newly identified cancer methylation biomarkers and evaluating their suitability for liquid biopsy testing are required to ensure continuous rapid progress. In this study, we followed a systematic approach beginning from the initial identification of markers in tumor DNA all the way through to analysis in patient plasma, with the added complexity of investigating three markers instead of one. At this stage of development, we consider the markers suitable for extensive laboratory-based testing, pushing the assay towards the required FDA standards.

Throughout this study, we have utilized a range of different data sources and techniques to evaluate the performance of the *ZNF154*, *TLX1*, and *GALR1* biomarkers individually or in combinations. Overall, we found a good correlation between the tissue-based and plasma-based analyses, despite the datasets being derived from DNA methylation array and whole genome bisulfite sequencing (WGBS) data, respectively. For example, in TCGA liver cancer tissue data, *ZNF154* alone could obtain a sensitivity of 87% and specificity of 98% with an AUC value at 0.92. The two-marker combination, *ZNF154* and *TLX1*, improved performance over *ZNF154* alone, increasing sensitivity to 92% with specificity still at 98%, and AUC at 0.95. The inclusion of *GALR1* did not improve results in either analysis. In the simulated blood samples with 10% ctDNA, *ZNF154* had an AUC of 0.84 in classifying tumor versus normal blood cell samples, which could be increased to 0.88 by adding *TLX1*. Again, the addition of *GALR1* did not improve the results markedly. Using the common threshold for classification of all tumor types as one disease, and then zooming in on the liver cancer cohort, *ZNF154* showed the best sensitivity of all single markers at 77%. As expected, the addition of *TLX1*, either with or without *GALR1*, improved the sensitivity to 80%. Through all these combinations the specificity emained constant with a range of 80 to 84%. Based on these results, we did not expand beyond three multi-cancer markers.

While the tissue-based analysis using TCGA data depended on the methylation levels at a single CpG probe expressed as a beta value (ranging from 0–1), analysis of whole genome bisulfite sequencing data added another level of complexity. These data provided information on the methylation density across reads overlapping a 1kb region, requiring more advanced computational tools for classification. We have previously developed such an application called the EpiClass tool, which takes into consideration both the methylation density (e.g., how many CpG sites in a given stretch of DNA is methylated) and the concentration of each of these differentially methylated epialleles [22]. Applying this technology on the sequenced plasma samples derived from patients with liver cancer or healthy controls, we found that *ZNF154* and *TLX1* performed with similar sensitivity (20% and 21%, respectively) when forcing the specificity to its maximum level (93% and 94%, respectively). If the two markers were combined in a single assay, such that a positive read-out for any of the two markers results in a positive test, the sensitivity increased to 33% along with a drop in specificity to 88%. Adding *GALR1* further improved sensitivity to 37% at a cost of 83% specificity. Following this sequential line of analyses from TCGA tissue data to actual plasma samples, it becomes evident how the dilute nature of ctDNA in liquid biopsies challenges assay performance. However, it should be noted that WGBS is likely not the optimal technology for detection of methylation at target loci in cfDNA samples. A natural consequence of the broadness of this approach (targeting the entire genome) is the relatively low coverage that results [33]. In addition, we consider this a pilot study, with provisional findings for early-stage cancer detection, as larger cohorts representing more tumor types are warranted to fully demonstrate the multi-cancer detection potential of our markers. Nonetheless, these data collectively point towards a strong performance when combining the *ZNF154* and *TLX1* markers into a single assay. While *GALR1* showed superiority in classifying TCGA tumor versus normal tissue derived from breast, kidney (KIRC) and lung (LUAD), it suffered from increased methylation levels in normal blood cells. Accordingly, *GALR1* had markedly worse performance at any simulated dilution level, reaching only 57% specificity in normal blood cell samples when ctDNA concentration was 10%. However, the utility of this marker cannot be ruled out, since the compromise in specificity led to *GALR1*, reaching the highest sensitivity as a multi-cancer assay within kidney, pancreatic, and lung adenocarcinoma.

We utilized the performance parameters, sensitivity, and specificity, obtained from diluted TCGA tumor methylation signals in a normal white blood cell background to estimate the putative positive predictive value (PPV) for our triple marker combination in a pan-cancer setting. PPV is a key metric used to evaluate harm-benefit in cancer testing, however it is most commonly calculated from large-scale clinical studies enrolling non-symptomatic individuals. As an example, we compared commercially available tests; the Cologuard^®^ has a PPV of 3.7% in detecting colorectal cancer [57], while low-dose CT has a PPV of 2.4% in detecting lung cancer and low-dose CT has a PPV of 2.4% in detecting lung cancer [58]. Pan-cancer detection platforms have yet to be evaluated for their performance in large asymptomatic cohorts of individuals. In the study by Klein and colleagues supporting Grail’s Galleri^®^ test, the authors extrapolate the PPV from their case control study using SEER cancer incidence rates in the 50–79 year age group [59]. With a stage I–IV sensitivity across 27 cancer classes of 51.5% and a specificity among healthy blood donors of 99.5%, they managed to obtain an estimated PPV of 44%. A recent announcement from Grail on the PATHFINDER study, which enrolled more than 6000 non-symptomatic individuals above 50 years old, revealed an actual PPV of 43.1% (https://grail.com/press-releases/grail-announces-final-results-from-the-pathfinder-multi-cancer-early-detection-screening-study-at-esmo-congress-2022/) (accessed on 11 September 2022).

A major factor affecting the PPV is the prevalence of the disease being detected in the tested population. In line with this, our data also demonstrate how grouping individual cancer types into one disease detected by a single assay drastically improves the PPV. Considering all seven possible marker combinations, we obtained an estimated PPV of 0.4% for colon and 0.3% for lung cancer, respectively. However, when considering all 14 cancer types combined, average PPV increased to 3.2%. Additionally, by setting a fixed specificity level at 95%, we managed to obtain a PPV of 8% for the triple marker assay. Comparing estimated PPV values head-to-head between studies should be conducted with the utmost caution. The study by Klein et al. is considered a clinical validation of pan-cancer testing of >4000 plasma samples from cancer patients and controls [59]. In the study presented here, the data derives from simulated blood samples based on 4050 tumor tissue samples and 2711 control white blood cell samples, yet still not actual plasma samples. Furthermore, the Galleri^®^ test is based on enrichment and sequencing of a methylation-specific panel covering 1,166,720 CpGs, whereas our technology focuses on reducing complexity in downstream analysis by targeting only three CpGs. While three markers represent a drastically reduced set, the benefits include a binary testing procedure producing only a yes or no answer. Other potential benefits include cost-effective applications and ease of use in laboratory testing. A three-marker test has been previously demonstrated to show high sensitivity and specificity in detecting colorectal cancer [60].

Utilizing TCGA data for pre-screening of potential cancer biomarkers has several limitations. First, a large number of TCGA samples analyzed here represented late-stage cancer diagnoses. Nonetheless, we were able to detect the presence of significant hypermethylation at the three biomarkers in the majority of stage I TCGA tumors compared to normal controls. Second, the undiluted TCGA methylation data represent an optimal sensitivity, specificity and AUC, (Table 1), which is unlikely to be reached in dilute plasma samples until technology improves. Third, TCGA methylation array data challenges biomarker performance by only providing array hybridization data on single CpG sites. This led to markedly decreased performance of all assays when simulating the concentration of tumor DNA down to 1%. A DNA-based liquid biopsy intended for early detection of cancer should preferably be able to detect the dilute nature of ctDNA relative to cell free DNA (cfDNA) where ratios of 1:1000 to 1:10,000 (ctDNA:cfDNA) are common [61,62,63,64]. Although some cancer patients present with increased concentrations of ctDNA [65], our analysis at 10% simulated tumor DNA concentration is far from the desirable clinical range. Thus, the use of single probe methylation array data will likely not suffice for early detection purposes. However, it still constitutes a powerful and highly relevant resource for assessing multi-cancer detection potential of several markers in a large dataset covering 14 tumor types in 4050 samples. Importantly, this multi-cancer panel covered tumor types where there is currently no screening option, such as pancreatic, bladder, and head and neck cancer. Combining detection of multiple cancers in one single assay puts a challenge on biomarker identification, but in turn provides tremendous clinical benefits. By targeting several tumor types, the incidence rates will aggregate and potentially allow low-prevalence cancers into broadly applicable screening programs, due to the boost in the positive predictive value (PPV) of a given test [66]. We note that our analysis does not constitute a diagnostic assay, and as such, the detection of cancer markers should be considered as a first step in pre-symptomatic testing. Notably, additional biomarkers continue to emerge to refine follow-up testing.

## 5. Conclusions

In summary, we present a three-marker combination of DNA methylation sites that, if further validated, could potentially be used as a first-pass cancer detection method, after which more specific biomarker testing procedures could be applied to reach a diagnosis. As an economical test, with a sufficiently high sensitivity and low false positive rate, this combination could be especially useful to complement existing screening methods, especially when screening for a given type of cancer does not currently exist.

## Figures and Tables

**Table 1:** Performance of the three-marker combination (*GALR1*, *TLX1*, and *ZNF154*) in classifying TCGA tumors.

Tumor Type ^1^	#Tumor Samples ^2^	#Normal Samples ^2^	Threshold ^3^	Sensitivity	Specificity	AUC ^4^
BLCA	201	20	0.93	90.5%	95.0%	0.967
BRCA	676	96	0.90	90.5%	95.8%	0.979
COAD	274	38	0.84	96.7%	100%	0.992
HNSC	426	50	0.80	98.6%	98.0%	0.996
KIRC	296	160	0.65	82.4%	95.6%	0.931
KIRP	156	45	0.70	73.7%	84.4%	0.859
LIHC	151	50	0.43	92.1%	98.0%	0.950
LUAD	437	32	0.98	97.3%	100%	0.999
LUSC	359	42	0.64	99.2%	100%	0.996
PAAD	65	9	0.58	98.5%	100%	0.991
PRAD	248	49	0.82	90.3%	91.8%	0.941
READ	96	7	0.50	100%	100%	1.000
STAD	260	2	0.50	100%	100%	1.000
UCEC	405	46	0.79	99.0%	100%	0.997

^1^ Tumor types are abbreviated as in Figure 1; ^2^ All methylation data were obtained from The Cancer Genome Atlas; ^3^ Probability threshold obtained by maximizing Youden’s Index after logistic regression and ROC curve analysis; ^4^ Abbreviations: AUC = Area Under the ROC Curve, ROC = Receiver Operating Characteristics.

**Table 2:** Performance of the three-marker combination (*GALR1*, *TLX1*, and *ZNF154*) in independent validation cohorts.

Tumor Type ^1^	Dataset	#Tumor Samples	#Normal Samples	Sensitivity	Specificity
BRCA	GSE37754, GSE66695, GSE69914	449	149	79.5%	94.0%
COAD	GSE53051	35	18	94.3%	83.3%
LUAD	GSE53051	9	11	100%	73%
PRAD	GSE112047	31	16	90.3%	100.0%

^1^ Tumor types are abbreviated as in Figure 1.

**Table 3:** Performance of the three-marker combination (*GALR1*, *TLX1*, and *ZNF154*) in plasma validation cohorts.

Tumor Type	Dataset	#Tumor Samples	#Normal Samples	Sensitivity ^1^	Specificity ^1^
Lung cancer	[33,34]	9	4	100% ^2^	100% ^2^
Hepatocellular carcinoma	[34,35]	30	36	37.3%	83.3%

^1^ Assay performance when thresholds were selected to maximize specificity. ^2^ Performance evaluated in training data only.

## Data Availability

Raw data are available through TCGA portal and Gene Expression Omnibus, GEO. Sample code will be made available at https://github.com/elnitskilab/Tumor_classification_bymethylation (accessed on 10 May 2023).

## Supplementary Materials

The following supporting information can be downloaded at: https://www.mdpi.com/article/10.3390/cancers15194826/s1, Figure S1: Schematic overview of ENCODE cell lines and their organ of origin; Figure S2: Heatmap of healthy and tumor cell line methylation; Figure S3: Receiver operating characteristic (ROC) curves showing classification performance of TCGA training cohorts. Figure S4: Hypermethylation at the three methylation probes in TCGA (training) vs. independent validation cohorts. Figure S5: Simulated dilution of tumor DNA at 1% in a background of WBC DNA; Figure S6: Sensitivity in detection of all tumor samples at 10% dilution level with specificity set to 95%; Figure S7: Breast tumor identification using the three biomarkers for bisulfite amplicon sequencing (biological replicates); Figure S8: Comparing single CpG analysis with average methylation across multiple CpGs in an amplicon; Figure S9: Heatmap illustrating the dual threshold of methylation density and epiallelic fraction; Figure S10: Example of AUC results for five-fold cross-validation; Table S1: Mean and median beta values for TCGA data Table S2: Logistic regression coefficients from analysis of TCGA tumor vs. normal tissue samples; Table S3: Measurements of accuracy from 10-fold cross-validation logistic regression modelling using tumor vs. blood samples; Table S4: Logistic regression coefficients from analysis of tumor vs. blood samples (10% ctDNA); Table S5: Tumor type incidence rates extracted from SEER Research Plus Data (November 2021 Submission); Table S6: Methylation density thresholds applied in EpiClass analysis of WGBS data from plasma; and density of reads in WGBS data; Table S7: Performance of *GALR1*, *TLX1*, and *ZNF154* in classifying TCGA tumors; Table S8: Performance of the three-marker combination (GALR1, TLX1, and ZNF154) in classifying TCGA tumors using cross-validation (training cohorts). Table S9: Performance of the three-marker combination (GALR1, TLX1, and ZNF154) in classifying TCGA tumors using cross-validation (validation cohorts). Table S10: Performance of *GALR1*, *TLX1*, and *ZNF154* in classifying tumor vs. normal samples in independent validation cohorts; Table S11: Performance of markers in simulated dilutions of tumor DNA into WBC DNA.

## Abbreviations

BLCA	bladder urothelial carcinoma;
BRCA	breast invasive carcinoma;
COAD	colon adenocarcinoma;
HNSC	head and neck squamous cell carcinoma;
KIRC	kidney renal clear cell carcinoma;
KIRP	kidney renal papillary cell carcinoma;
LIHC	liver hepatocellular carcinoma;
LUAD	lung adenocarcinoma; LUSC, lung squamous cell carcinoma;
PAAD	pancreatic adenocarcinoma;
PRAD	prostate adenocarcinoma;
READ	rectum adenocarcinoma;
STAD	stomach adenocarcinoma;
UCEC	uterine corpus endometrioid carcinoma.
