# Supplementary material for: Evaluating Stacked Methylation Markers for Blood-Based Multicancer Detection
Source: Cancers (Basel). 2023 Oct 1;15(19):4826. doi: 10.3390/cancers15194826 (PMC10571530; doi:10.3390/cancers15194826)
Supplement: Supplementary file 1 [file cancers-15-04826-s001.zip › Supplementary Figures.pptx]

## Slide 1
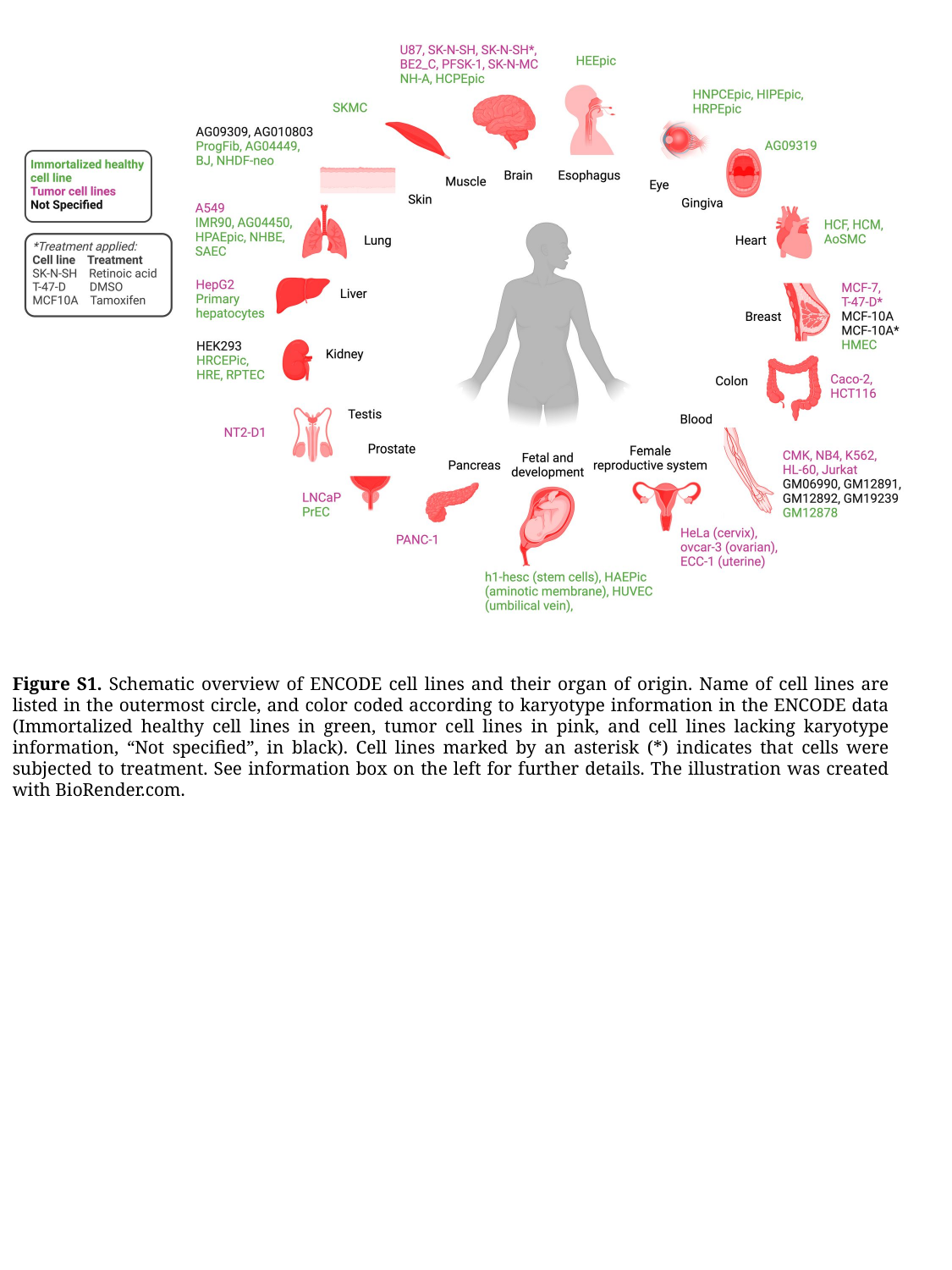

Figure S1. Schematic overview of ENCODE cell lines and their organ of origin. Name of cell lines are listed in the outermost circle, and color coded according to karyotype information in the ENCODE data (Immortalized healthy cell lines in green, tumor cell lines in pink, and cell lines lacking karyotype information, “Not specified”, in black). Cell lines marked by an asterisk (*) indicates that cells were subjected to treatment. See information box on the left for further details. The illustration was created with BioRender.com.

## Slide 2
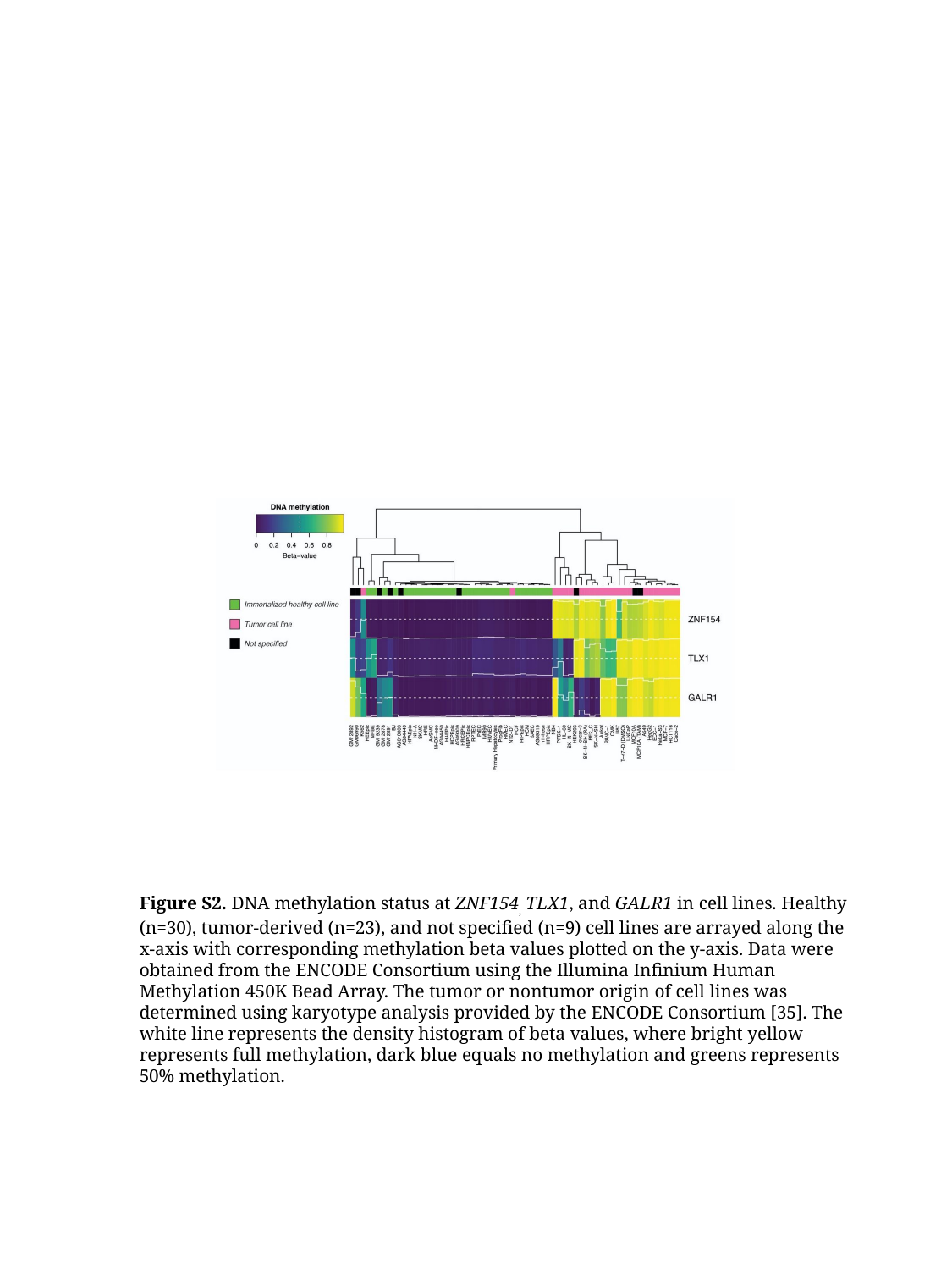

Figure S2. DNA methylation status at ZNF154, TLX1, and GALR1 in cell lines. Healthy (n=30), tumor-derived (n=23), and not specified (n=9) cell lines are arrayed along the x-axis with corresponding methylation beta values plotted on the y-axis. Data were obtained from the ENCODE Consortium using the Illumina Infinium Human Methylation 450K Bead Array. The tumor or nontumor origin of cell lines was determined using karyotype analysis provided by the ENCODE Consortium [35]. The white line represents the density histogram of beta values, where bright yellow represents full methylation, dark blue equals no methylation and greens represents 50% methylation.

## Slide 3
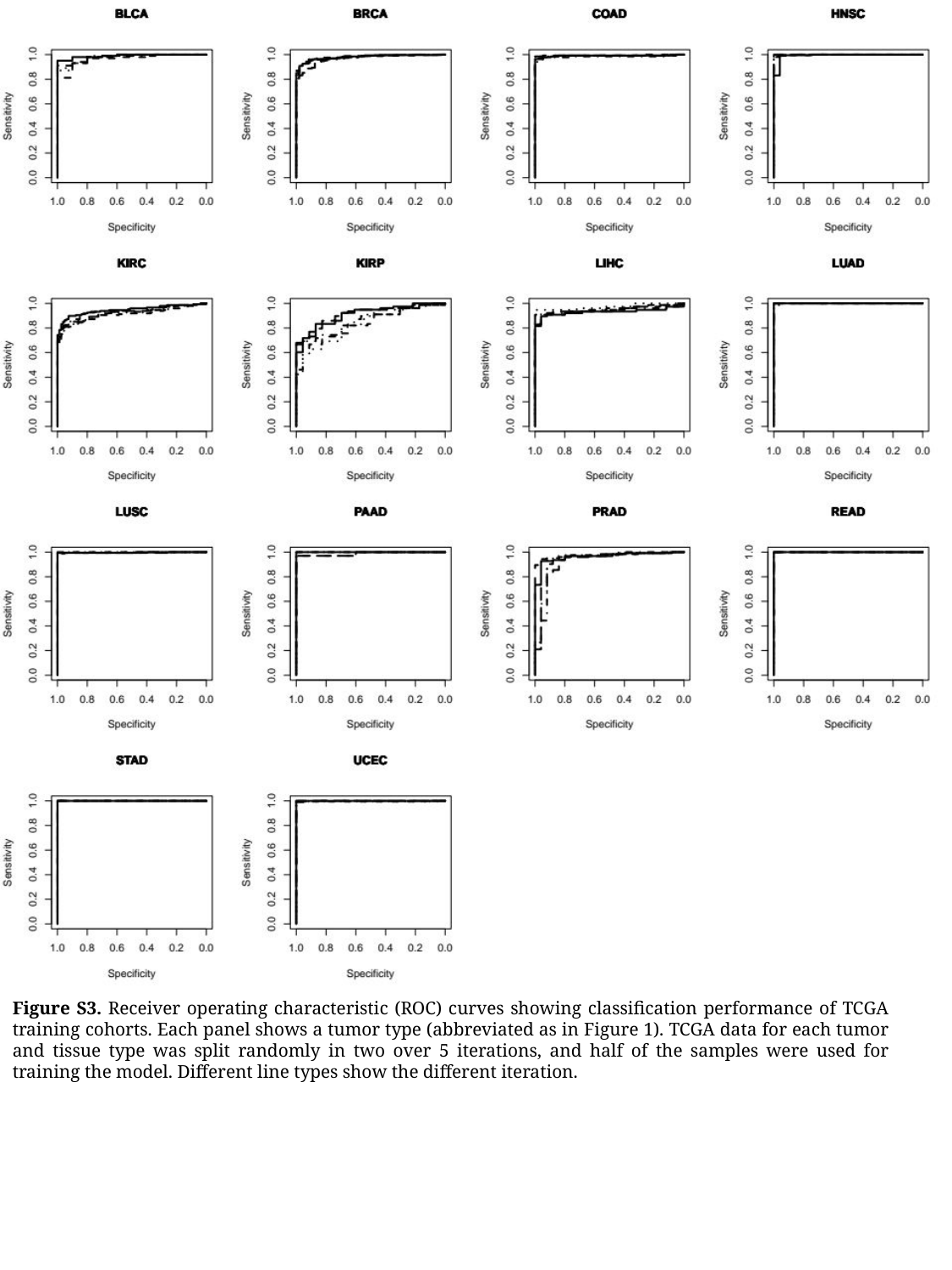

Figure S3. Receiver operating characteristic (ROC) curves showing classification performance of TCGA training cohorts. Each panel shows a tumor type (abbreviated as in Figure 1). TCGA data for each tumor and tissue type was split randomly in two over 5 iterations, and half of the samples were used for training the model. Different line types show the different iteration.

## Slide 4
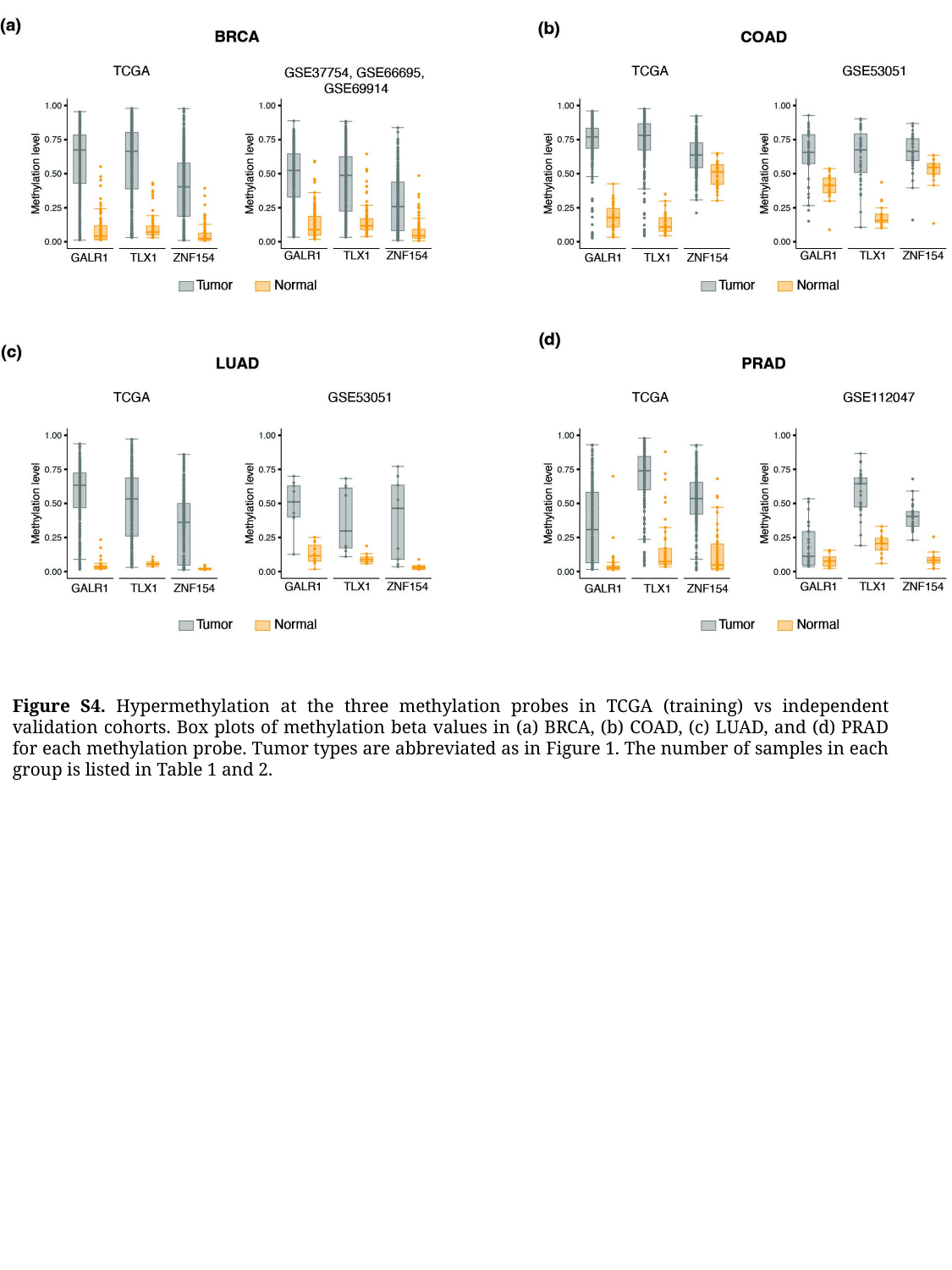

Figure S4. Hypermethylation at the three methylation probes in TCGA (training) vs independent validation cohorts. Box plots of methylation beta values in (a) BRCA, (b) COAD, (c) LUAD, and (d) PRAD for each methylation probe. Tumor types are abbreviated as in Figure 1. The number of samples in each group is listed in Table 1 and 2.

## Slide 5
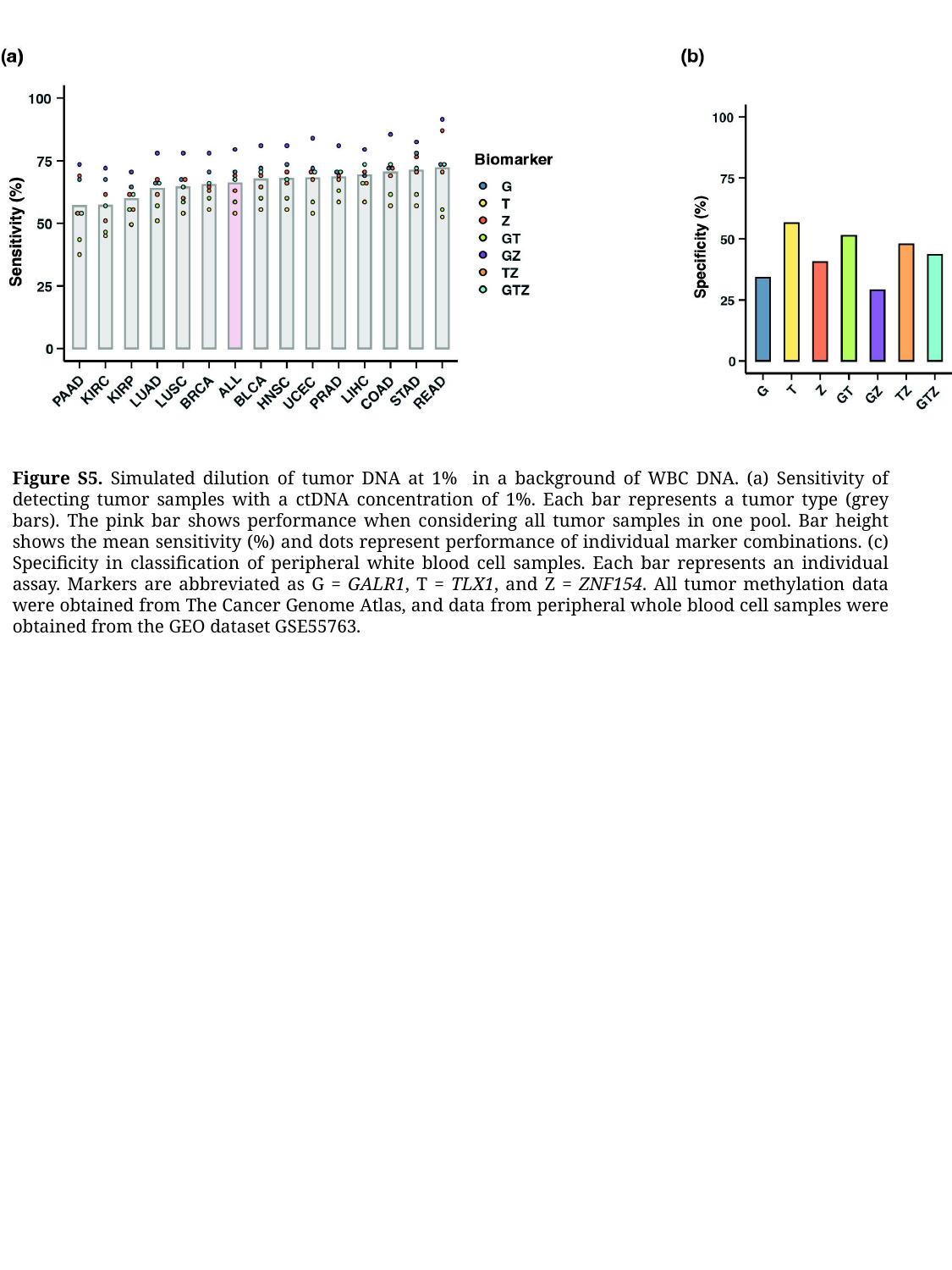

Figure S5. Simulated dilution of tumor DNA at 1% in a background of WBC DNA. (a) Sensitivity of detecting tumor samples with a ctDNA concentration of 1%. Each bar represents a tumor type (grey bars). The pink bar shows performance when considering all tumor samples in one pool. Bar height shows the mean sensitivity (%) and dots represent performance of individual marker combinations. (c) Specificity in classification of peripheral white blood cell samples. Each bar represents an individual assay. Markers are abbreviated as G = GALR1, T = TLX1, and Z = ZNF154. All tumor methylation data were obtained from The Cancer Genome Atlas, and data from peripheral whole blood cell samples were obtained from the GEO dataset GSE55763.

## Slide 6
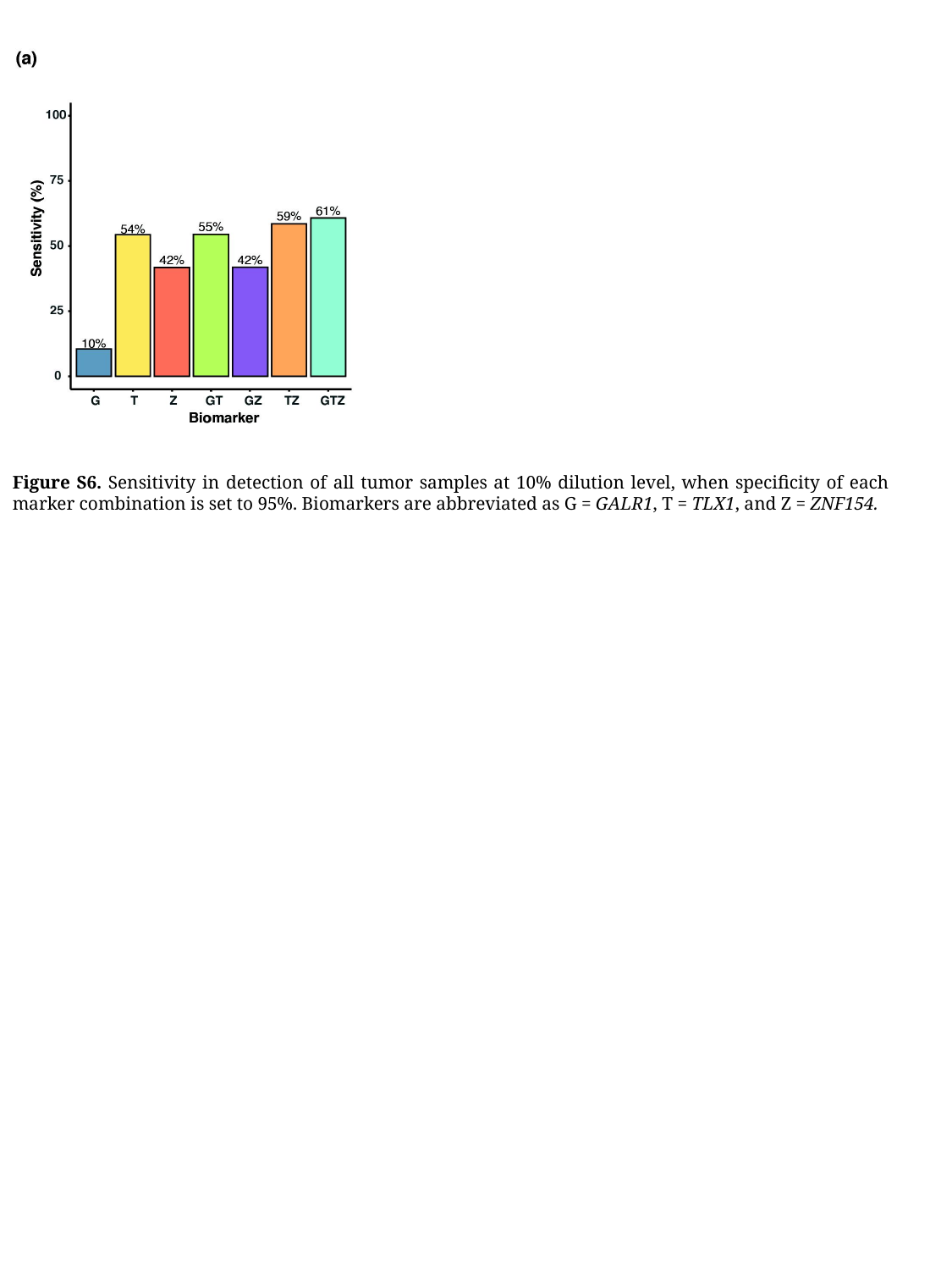

Figure S6. Sensitivity in detection of all tumor samples at 10% dilution level, when specificity of each marker combination is set to 95%. Biomarkers are abbreviated as G = GALR1, T = TLX1, and Z = ZNF154.

## Slide 7
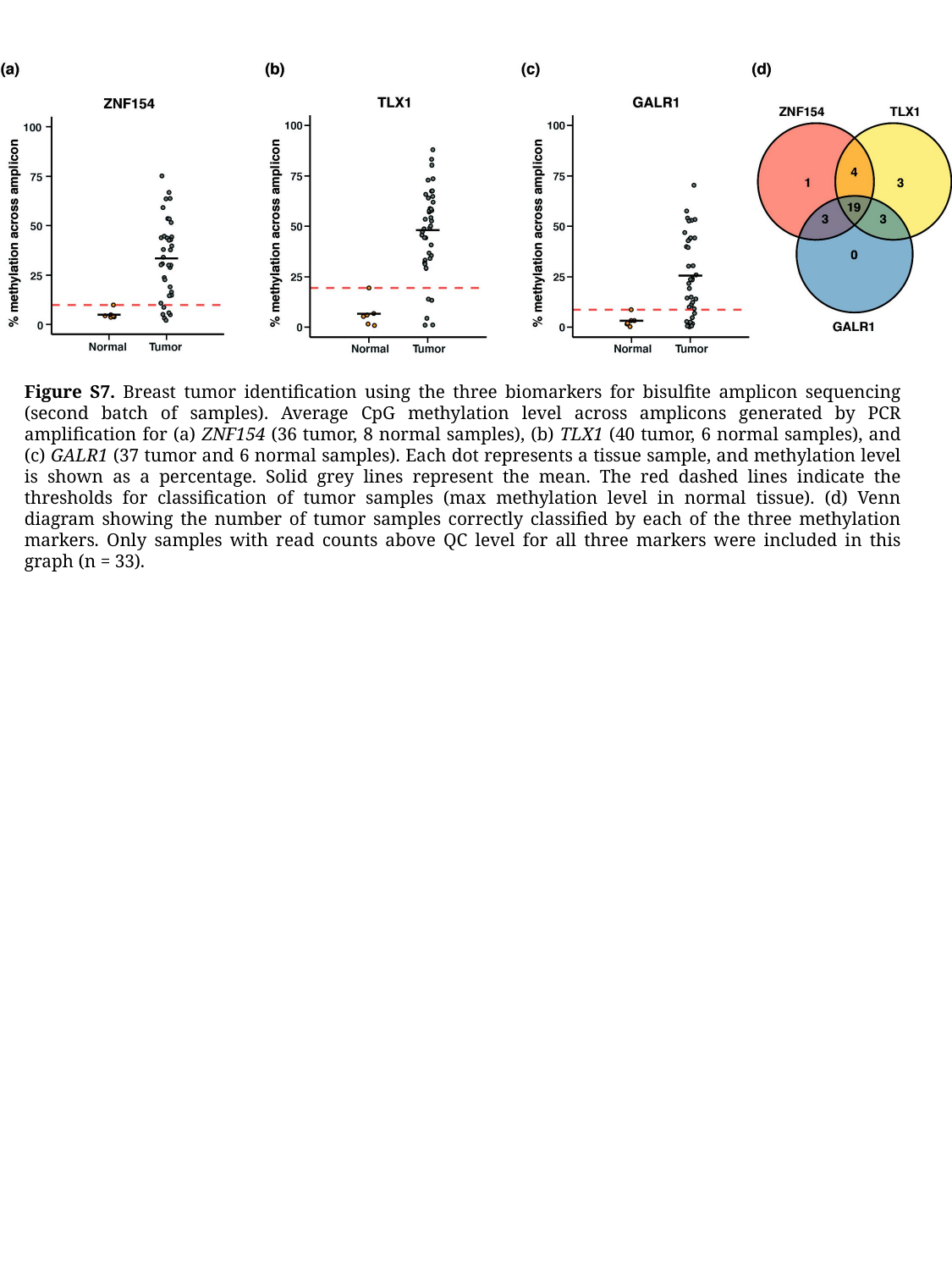

Figure S7. Breast tumor identification using the three biomarkers for bisulfite amplicon sequencing (second batch of samples). Average CpG methylation level across amplicons generated by PCR amplification for (a) ZNF154 (36 tumor, 8 normal samples), (b) TLX1 (40 tumor, 6 normal samples), and (c) GALR1 (37 tumor and 6 normal samples). Each dot represents a tissue sample, and methylation level is shown as a percentage. Solid grey lines represent the mean. The red dashed lines indicate the thresholds for classification of tumor samples (max methylation level in normal tissue). (d) Venn diagram showing the number of tumor samples correctly classified by each of the three methylation markers. Only samples with read counts above QC level for all three markers were included in this graph (n = 33).

## Slide 8
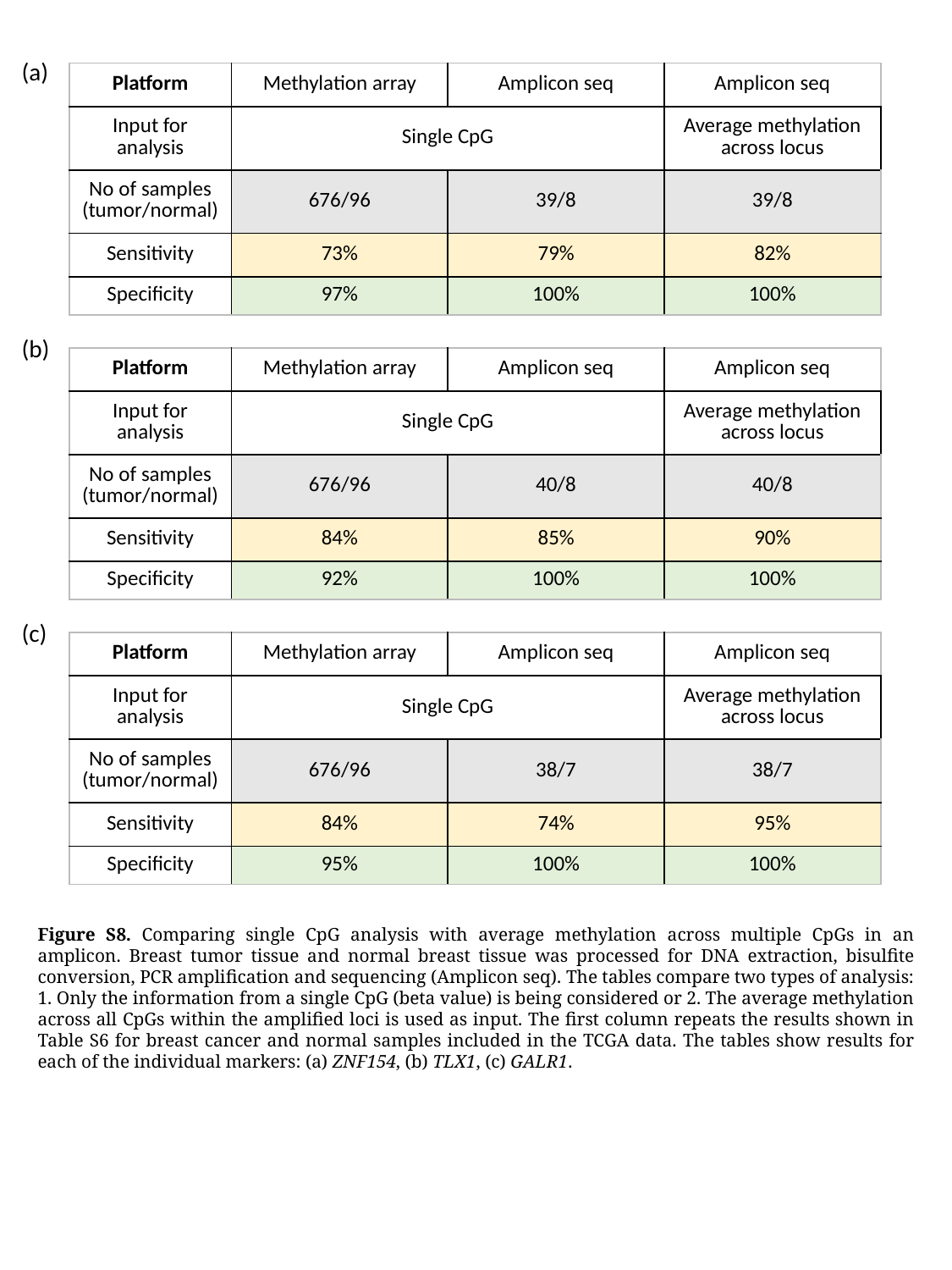

(a)
| Platform | Methylation array | Amplicon seq | Amplicon seq |
| --- | --- | --- | --- |
| Input for analysis | Single CpG | | Average methylation across locus |
| No of samples (tumor/normal) | 676/96 | 39/8 | 39/8 |
| Sensitivity | 73% | 79% | 82% |
| Specificity | 97% | 100% | 100% |
(b)
| Platform | Methylation array | Amplicon seq | Amplicon seq |
| --- | --- | --- | --- |
| Input for analysis | Single CpG | | Average methylation across locus |
| No of samples (tumor/normal) | 676/96 | 40/8 | 40/8 |
| Sensitivity | 84% | 85% | 90% |
| Specificity | 92% | 100% | 100% |
(c)
| Platform | Methylation array | Amplicon seq | Amplicon seq |
| --- | --- | --- | --- |
| Input for analysis | Single CpG | | Average methylation across locus |
| No of samples (tumor/normal) | 676/96 | 38/7 | 38/7 |
| Sensitivity | 84% | 74% | 95% |
| Specificity | 95% | 100% | 100% |
Figure S8. Comparing single CpG analysis with average methylation across multiple CpGs in an amplicon. Breast tumor tissue and normal breast tissue was processed for DNA extraction, bisulfite conversion, PCR amplification and sequencing (Amplicon seq). The tables compare two types of analysis: 1. Only the information from a single CpG (beta value) is being considered or 2. The average methylation across all CpGs within the amplified loci is used as input. The first column repeats the results shown in Table S6 for breast cancer and normal samples included in the TCGA data. The tables show results for each of the individual markers: (a) ZNF154, (b) TLX1, (c) GALR1.

## Slide 9
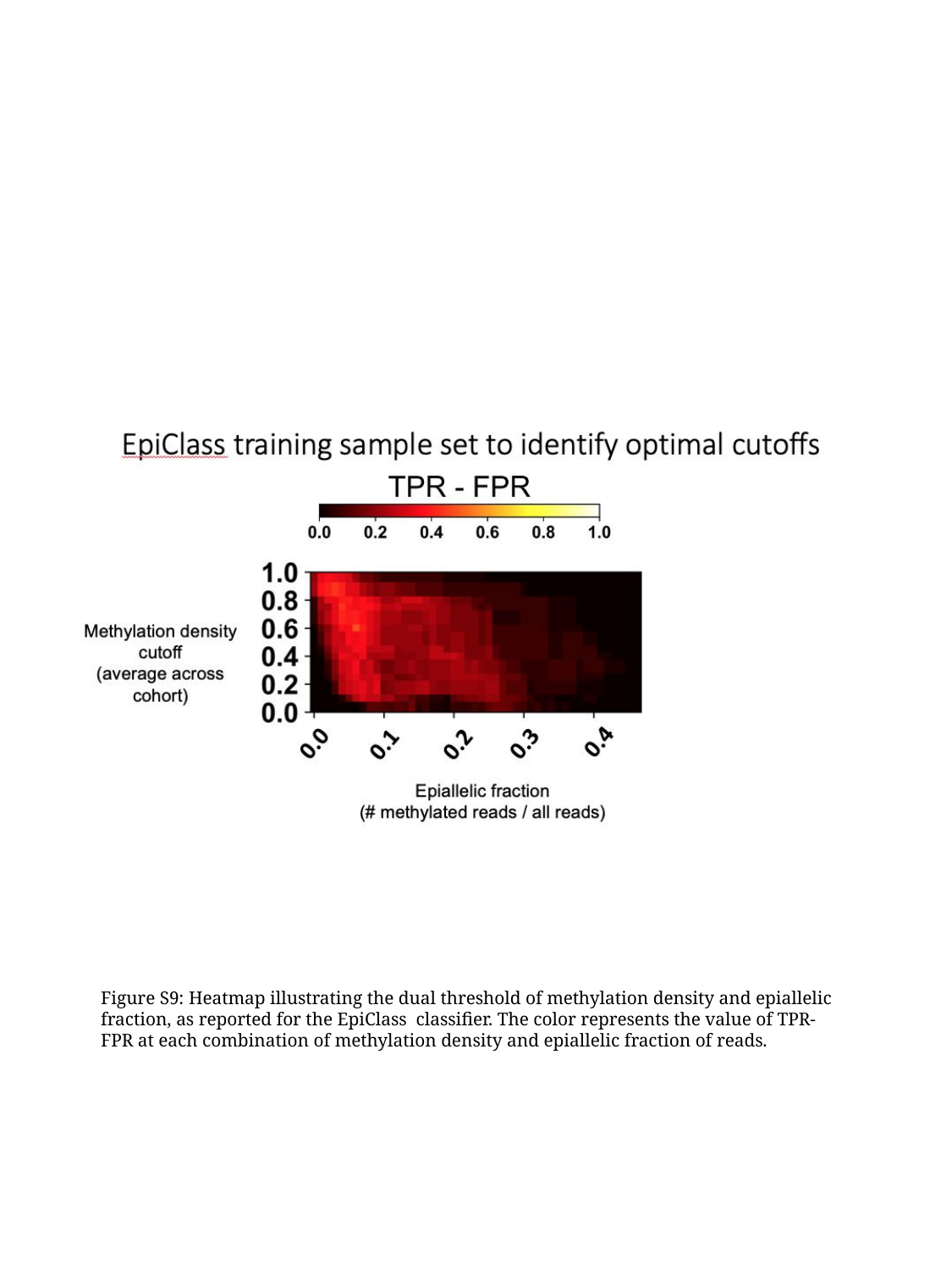

Figure S9: Heatmap illustrating the dual threshold of methylation density and epiallelic fraction, as reported for the EpiClass classifier. The color represents the value of TPR-FPR at each combination of methylation density and epiallelic fraction of reads.

## Slide 10
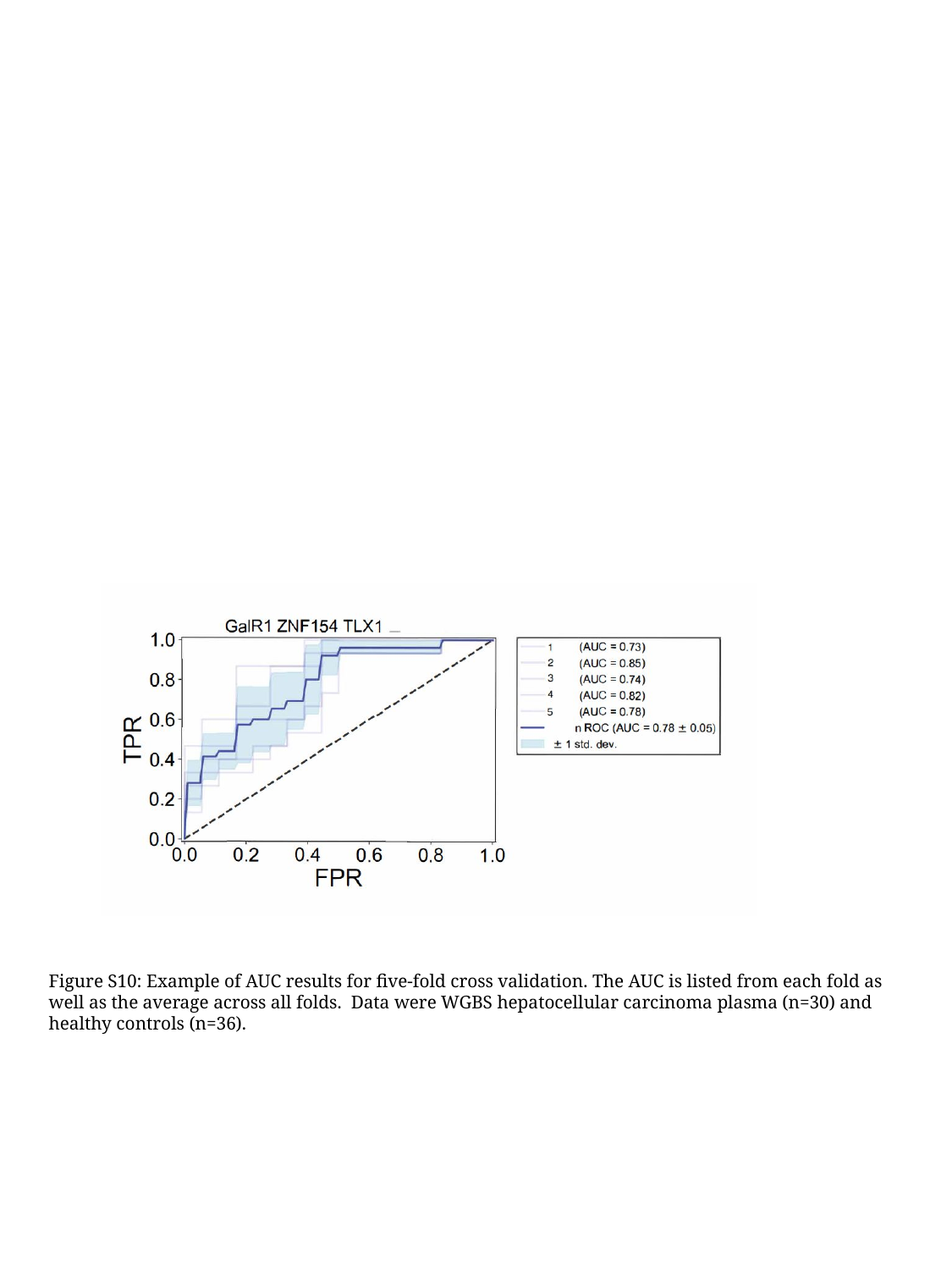

Figure S10: Example of AUC results for five-fold cross validation. The AUC is listed from each fold as well as the average across all folds. Data were WGBS hepatocellular carcinoma plasma (n=30) and healthy controls (n=36).
