# Supplementary material for: Evaluating Stacked Methylation Markers for Blood-Based Multicancer Detection
Source: Cancers (Basel). 2023 Oct 1;15(19):4826. doi: 10.3390/cancers15194826 (PMC10571530; doi:10.3390/cancers15194826)
Supplement: Supplementary file 1 [file cancers-15-04826-s001.zip › Table S11.pdf]

Table S11. Performance of markers in simulated dilutions of tumor DNA into WBC DNA

Biomarker	ctDNA fraction[a]	AUC	Threshold[b]	Specificity	Sensitivity														
					ALL[c]	*BLCA*	*BRCA*	*COAD*	*HNSC*	*KIRC*	*KRIP*	*LIHC*	*LUAD*	*LUSC*	*PAAD*	*PRAD*	*READ*	*STAD*	*UCEC*
G		0.523	0.49	34%	70%	73%	70%	72%	73%	67%	64%	70%	66%	68%	68%	70%	73%	77%	72%
T		0.559	0.50	57%	54%	56%	55%	57%	55%	45%	49%	59%	51%	53%	37%	58%	53%	57%	53%
Z		0.554	0.48	41%	69%	69%	65%	72%	71%	61%	62%	71%	67%	68%	69%	69%	86%	77%	71%
GT	0.01	0.559	0.49	51%	58%	60%	59%	62%	61%	47%	55%	66%	56%	58%	43%	63%	56%	61%	58%
GZ		0.554	0.47	29%	80%	81%	78%	86%	81%	72%	70%	80%	78%	79%	74%	81%	92%	83%	84%
TZ		0.566	0.48	48%	64%	64%	63%	70%	66%	51%	56%	66%	61%	60%	54%	68%	71%	70%	67%
GTZ		0.568	0.48	44%	67%	70%	66%	73%	67%	57%	61%	73%	67%	64%	54%	70%	73%	72%	70%
G		0.700	0.45	57%	75%	87%	79%	89%	85%	53%	42%	57%	77%	72%	69%	63%	85%	91%	73%
T		0.848	0.53	85%	74%	69%	75%	93%	85%	26%	28%	70%	69%	80%	49%	89%	95%	90%	80%
Z		0.835	0.48	80%	76%	88%	67%	97%	92%	34%	32%	77%	61%	77%	57%	84%	99%	91%	98%
GT	0.1	0.848	0.53	86%	73%	68%	75%	93%	85%	25%	28%	70%	69%	80%	49%	88%	95%	90%	80%
GZ		0.836	0.51	81%	75%	88%	65%	97%	91%	32%	29%	77%	61%	76%	55%	83%	99%	88%	98%
TZ		0.881	0.48	83%	79%	85%	77%	95%	93%	32%	38%	80%	71%	84%	54%	90%	98%	94%	94%
GTZ		0.881	0.49	84%	79%	82%	77%	96%	92%	30%	37%	80%	70%	86%	55%	92%	98%	92%	95%
G		0.815	0.56	87%	70%	88%	77%	94%	88%	39%	14%	36%	76%	65%	68%	45%	81%	95%	65%
T		0.897	0.58	96%	76%	71%	79%	96%	90%	24%	23%	68%	74%	83%	57%	93%	97%	95%	80%
Z		0.900	0.65	97%	76%	93%	66%	100%	96%	23%	21%	77%	61%	81%	52%	90%	100%	94%	100%
GT	0.25	0.911	0.52	94%	79%	76%	83%	96%	92%	27%	23%	69%	79%	85%	68%	93%	97%	97%	81%
GZ		0.915	0.65	96%	77%	93%	70%	100%	96%	24%	21%	74%	65%	82%	54%	86%	100%	95%	99%
TZ		0.939	0.51	95%	85%	90%	87%	100%	99%	29%	28%	86%	83%	92%	69%	95%	100%	99%	99%
GTZ		0.939	0.61	97%	83%	87%	84%	99%	98%	25%	25%	81%	78%	91%	60%	94%	100%	97%	99%

G		0.832	0.58	95%	71%	89%	80%	95%	90%	38%	11%	34%	81%	69%	75%	46%	81%	97%	63%
T		0.903	0.58	99%	78%	74%	83%	96%	90%	26%	24%	70%	80%	84%	60%	95%	97%	96%	83%
Z		0.904	0.52	98%	82%	94%	76%	100%	99%	33%	29%	82%	68%	89%	71%	94%	100%	98%	100%
GT	0.5	0.924	0.53	97%	82%	87%	86%	98%	98%	31%	22%	70%	87%	89%	72%	94%	99%	97%	83%
GZ		0.933	0.48	96%	86%	95%	87%	100%	100%	38%	26%	79%	82%	92%	80%	94%	100%	99%	100%
TZ		0.947	0.54	99%	87%	94%	91%	100%	100%	34%	29%	88%	87%	94%	75%	96%	100%	99%	100%
GTZ		0.949	0.53	99%	88%	94%	91%	100%	100%	35%	28%	87%	89%	94%	75%	96%	100%	100%	99%
G		0.818	0.54	96%	73%	92%	84%	95%	92%	39%	12%	36%	83%	71%	77%	50%	83%	97%	63%
T		0.899	0.53	99%	80%	76%	84%	97%	91%	29%	28%	70%	82%	86%	65%	95%	97%	96%	83%
Z		0.895	0.53	99%	82%	95%	76%	100%	99%	35%	31%	82%	69%	89%	74%	94%	100%	98%	100%
GT	0.75	0.921	0.44	97%	86%	93%	90%	99%	99%	40%	26%	71%	93%	92%	80%	95%	99%	98%	86%
GZ		0.930	0.50	98%	88%	96%	91%	100%	100%	41%	26%	80%	87%	94%	83%	94%	100%	99%	100%
TZ		0.946	0.46	99%	89%	96%	93%	100%	100%	39%	33%	89%	90%	95%	82%	96%	100%	99%	100%
GTZ		0.948	0.51	99%	89%	96%	94%	100%	100%	39%	29%	89%	92%	95%	82%	96%	100%	100%	100%
G		0.812	0.58	98%	73%	90%	83%	95%	91%	39%	12%	36%	83%	69%	75%	49%	83%	97%	63%
T		0.895	0.49	99%	81%	77%	84%	97%	91%	31%	29%	71%	85%	86%	68%	95%	97%	96%	83%
Z		0.888	0.47	99%	83%	95%	77%	100%	99%	39%	31%	83%	70%	90%	74%	94%	100%	98%	100%
GT	0.9	0.919	0.44	98%	86%	93%	91%	99%	99%	41%	26%	72%	93%	92%	80%	96%	99%	98%	86%
GZ		0.927	0.49	99%	88%	96%	91%	100%	100%	43%	26%	81%	88%	94%	82%	94%	100%	99%	100%
TZ		0.944	0.42	99%	90%	97%	94%	100%	100%	41%	35%	90%	92%	95%	83%	96%	100%	99%	100%
GTZ		0.947	0.38	99%	90%	97%	95%	100%	100%	43%	34%	89%	93%	96%	85%	96%	100%	100%	100%
G		0.810	0.54	98%	74%	92%	84%	95%	92%	40%	12%	36%	84%	71%	77%	51%	83%	97%	63%
T		0.893	0.47	99%	81%	77%	85%	97%	91%	32%	30%	72%	85%	86%	68%	95%	97%	96%	83%
Z		0.884	0.44	99%	83%	95%	78%	100%	99%	40%	31%	84%	70%	90%	75%	95%	100%	99%	100%
GT	1	0.918	0.41	98%	87%	93%	92%	99%	99%	42%	28%	72%	93%	93%	85%	96%	99%	98%	86%
GZ		0.927	0.46	99%	88%	97%	91%	100%	100%	45%	26%	81%	89%	95%	85%	94%	100%	99%	100%
TZ		0.943	0.41	99%	90%	97%	94%	100%	100%	43%	35%	90%	92%	95%	83%	96%	100%	99%	100%
GTZ		0.946	0.44	99%	90%	97%	95%	100%	100%	42%	33%	89%	93%	96%	83%	96%	100%	100%	100%

[a] *Methylation value from TCGA tumor samples were computationally diluted at different concentrations into the methylation value of white blood cell DNA, to simulate the circulating tumor DNA (ctDNA) found in plasma.*
[b] *Probability threshold obtained by applying Youden's Index after logistic regression and ROC curve analysis*
[c] *Tumor samples from all 14 TCGA tumor types combined into one cohort.*
Abbreviations: G = GALR1, T = TLX1, Z = ZNF154, AUC = Area Under the ROC curve, ROC = Receiver Operating Characteristics, BLCA = bladder urothelial carcinoma, BRCA = breast invasive carcinoma, COAD = colon adenocarcinoma, HNSC = head and neck squamous cell carcinoma, KIRC = kidney renal clear cell carcinoma, KIRP = kidney renal papillary cell carcinoma, LIHC = liver hepatocellular carcinoma, LUAD = lung adenocarcinoma, LUSC = lung squamous cell carcinoma, PAAD = pancreatic adenocarcinoma, PRAD = prostate adenocarcinoma, READ = rectum adenocarcinoma, STAD = stomach adenocarcinoma, UCEC = uterine corpus endometrioid carcinoma.
