# Supplementary material for: Evaluating Stacked Methylation Markers for Blood-Based Multicancer Detection
Source: Cancers (Basel). 2023 Oct 1;15(19):4826. doi: 10.3390/cancers15194826 (PMC10571530; doi:10.3390/cancers15194826)
Supplement: Supplementary file 1 [file cancers-15-04826-s001.zip › Table S10.pdf]

Table S10. Performance of GALR1, TLX1, and ZNF154 in independent validation cohorts

Type[a]	Combination	TP	FP	TN	FN	Sensitivity	Specificity	Dataset
BRCA	GALR1	353	11	138	96	78.6%	92.6%	GSE37754, GSE66695, GSE69914
	TLX1	363	27	122	86	80.8%	81.9%	
	ZNF154	248	12	137	201	55.2%	91.9%	
	GALR1 + TLX1	386	13	136	63	86.0%	91.3%	
	GALR1 + ZNF154	336	8	141	113	74.8%	94.6%	
	TLX1 + ZNF154	332	9	140	117	73.9%	94.0%	
	GALR1 + TLX1 + ZNF154	357	9	140	92	79.5%	94.0%	
COAD	GALR1	28	6	12	7	80.0%	66.7%	GSE53051
	TLX1	31	1	17	4	88.6%	94.4%	
	ZNF154	26	4	14	9	74.3%	77.8%	
	GALR1 + TLX1	33	3	15	2	94.3%	83.3%	
	GALR1 + ZNF154	29	7	11	6	82.9%	61.1%	
	TLX1 + ZNF154	30	1	17	5	85.7%	94.4%	
	GALR1 + TLX1 + ZNF154	33	3	15	2	94.3%	83.3%	
LUAD	GALR1	9	6	5	0	100.0%	45.5%	GSE53051
	TLX1	9	6	5	0	100.0%	45.5%	
	ZNF154	8	1	10	1	88.9%	90.9%	
	GALR1 + TLX1	9	5	6	0	100.0%	54.5%	
	GALR1 + ZNF154	9	3	8	0	100.0%	72.7%	
	TLX1 + ZNF154	9	2	9	0	100.0%	81.8%	
	GALR1 + TLX1 + ZNF154	9	3	8	0	100.0%	72.7%	
PRAD	GALR1	15	4	12	16	48.4%	75.0%	GSE112047
	TLX1	28	0	16	3	90.3%	100.0%	
	ZNF154	27	0	16	4	87.1%	100.0%	
	GALR1 + TLX1	29	1	15	2	93.6%	93.8%	
	GALR1 + ZNF154	28	1	15	3	90.3%	93.8%	
	TLX1 + ZNF154	28	0	16	3	90.3%	100.0%	
	GALR1 + TLX1 + ZNF154	28	0	16	3	90.3%	100.0%	

[a] *Tumor types are abbreviated as in Figure 1*

Abbreviations: TP = True Positive, FP = False Positive, TN = True Negative, FN = False Negative,
