# Supplementary material for: Evaluating Stacked Methylation Markers for Blood-Based Multicancer Detection
Source: Cancers (Basel). 2023 Oct 1;15(19):4826. doi: 10.3390/cancers15194826 (PMC10571530; doi:10.3390/cancers15194826)
Supplement: Supplementary file 1 [file cancers-15-04826-s001.zip › Table S9.pdf]

Table S9. Performance of the three-marker combination (*GALR1*, *TLX1*, and *ZNF154*) in classifying TCGA tumors using cross validation (validation cohorts)

Type[a]	Tumor samples	Normal samples	Sensitivity		Specificity	
			Mean[b]	*SD*	*Mean*[b]	*SD*
BLCA	100	10	92.4%	2.1	80.0%	12.2
BRCA	338	48	92.7%	2.3	89.2%	6.0
COAD	137	19	95.6%	1.3	98.9%	2.4
HNSC	213	25	99.0%	0.6	94.4%	2.2
KIRC	148	80	84.9%	2.7	91.3%	3.2
KIRP	78	22	72.3%	14.7	71.8%	23.7
LIHC	75	25	92.3%	2.6	95.2%	5.2
LUAD	218	16	98.5%	1.5	88.8%	10.3
LUSC	179	21	98.9%	0.9	94.3%	2.1
PAAD*	32	4	95.6%	5.2	90.0%	13.7
PRAD	124	24	90.9%	1.7	83.3%	5.1
READ*	48	3	97.5%	2.3	100.0%	0.0
STAD*	130	1	98.6%	1.0	20.0%	44.7
UCEC	202	23	98.6%	0.5	94.8%	7.1

[a] Tumor types are abbreviated as in Figure 1
[b] TCGA datasets were randomly split in two five times and used as training/validation cohorts. The mean and SD report the average performance across the five iterations.

** Due to <10 samples in either the tumor or normal cohort, the performance results should be interpreted with caution*

Abbreviations: SD = Standard Deviation
