# Supplementary material for: Evaluating Stacked Methylation Markers for Blood-Based Multicancer Detection
Source: Cancers (Basel). 2023 Oct 1;15(19):4826. doi: 10.3390/cancers15194826 (PMC10571530; doi:10.3390/cancers15194826)
Supplement: Supplementary file 1 [file cancers-15-04826-s001.zip › Table S8.pdf]

Table S8. Performance of the three-marker combination (*GALR1*, *TLX1*, and *ZNF154*) in classifying TCGA tumors using cross validation (training cohorts)

Type[a]	Tumor samples	Normal samples	Threshold[b]		Sensitivity		Specificity		AUC	
			Mean[c]	*SD*	*Mean[c]*	*SD*	*Mean[c]*	*SD*	*Mean[c]*	*SD*
BLCA	101	10	0.89	0.08	91.3%	3.3	98.0%	4.5	0.978	0.009
BRCA	338	48	0.84	0.10	91.6%	2.9	95.8%	2.1	0.979	0.006
COAD	137	19	0.80	0.06	96.8%	0.8	100.0%	0.0	0.990	0.005
HNSC	213	25	0.59	0.13	99.7%	0.4	99.2%	1.8	0.998	0.002
KIRC	148	80	0.52	0.06	85.1%	3.4	93.5%	1.9	0.930	0.013
KIRP	78	23	0.71	0.11	75.9%	10.5	87.9%	10.8	0.870	0.016
LIHC	76	25	0.43	0.09	91.6%	2.7	96.8%	1.8	0.942	0.018
LUAD	219	16	0.63	0.18	99.5%	0.7	100.0%	0.0	1.000	0.001
LUSC	180	21	0.56	0.06	99.4%	0.4	99.0%	2.1	0.992	0.011
PAAD*	33	5	0.56	0.09	98.8%	1.6	100.0%	0.0	0.993	0.011
PRAD	124	25	0.73	0.11	93.2%	1.5	92.0%	4.9	0.950	0.016
READ*	48	4	0.50	0.00	100.0%	0.0	100.0%	0.0	1.000	0.000
STAD*	130	1	0.50	0.00	100.0%	0.0	100.0%	0.0	1.000	0.000
UCEC	203	23	0.59	0.14	99.8%	0.3	100.0%	0.0	1.000	0.000

[a] Tumor types are abbreviated as in Figure 1
[b] Probability threshold obtained by applying Youden's Index after logistic regression and ROC curve analysis
[c] TCGA datasets were randomly split in two five times and used as training/validation cohorts. The mean and SD report the average performance across the five iterations.

** Due to <10 samples in either the tumor or normal cohort, the performance results should be interpreted with caution*

Abbreviations: SD = Standard Deviation, AUC = Area Under the ROC Curve, ROC = Receiver Operating Characteristics
