# Supplementary material for: Evaluating Stacked Methylation Markers for Blood-Based Multicancer Detection
Source: Cancers (Basel). 2023 Oct 1;15(19):4826. doi: 10.3390/cancers15194826 (PMC10571530; doi:10.3390/cancers15194826)
Supplement: Supplementary file 1 [file cancers-15-04826-s001.zip › Table S7.pdf]

Table S7. Performance of GALR1, TLX1, and ZNF154 in classifying TCGA tumors

Type[a]	Combination	Threshold[b]	TP	FP	TN	FN	Sensitivity	Specificity	AUC
BLCA	GALR1	0.97	163	1	19	38	81.1%	95.0%	0.934
	TLX1	0.92	143	1	19	58	71.1%	95.0%	0.865
	ZNF154	0.96	174	0	20	27	86.6%	100.0%	0.961
	GALR1 + TLX1	0.76	187	3	17	14	93.0%	85.0%	0.944
	GALR1 + ZNF154	0.95	178	1	19	23	88.6%	95.0%	0.967
	TLX1 + ZNF154	0.91	184	1	19	17	91.5%	95.0%	0.968
	GALR1 + TLX1 + ZNF154	0.93	182	1	19	19	90.5%	95.0%	0.967
BRCA	GALR1	0.88	570	5	91	106	84.3%	94.8%	0.935
	TLX1	0.78	565	8	88	111	83.6%	91.7%	0.906
	ZNF154	0.91	496	3	93	180	73.4%	96.9%	0.865
	GALR1 + TLX1	0.83	612	6	90	64	90.5%	93.8%	0.962
	GALR1 + ZNF154	0.93	605	4	92	71	89.5%	95.8%	0.967
	TLX1 + ZNF154	0.92	582	3	93	94	86.1%	96.9%	0.964
	GALR1 + TLX1 + ZNF154	0.90	612	4	92	64	90.5%	95.8%	0.979
COAD	GALR1	0.85	259	0	38	15	94.5%	100.0%	0.965
	TLX1	0.81	260	0	38	14	94.9%	100.0%	0.973
	ZNF154	0.91	176	3	35	98	64.2%	92.1%	0.812
	GALR1 + TLX1	0.85	264	0	38	10	96.4%	100.0%	0.992
	GALR1 + ZNF154	0.91	257	0	38	17	93.8%	100.0%	0.979
	TLX1 + ZNF154	0.88	257	0	38	17	93.8%	100.0%	0.978
	GALR1 + TLX1 + ZNF154	0.84	265	0	38	9	96.7%	100.0%	0.992
HNSC	GALR1	0.94	370	1	49	56	86.9%	98.0%	0.945
	TLX1	0.88	378	1	49	48	88.7%	98.0%	0.934
	ZNF154	0.66	418	2	48	8	98.1%	96.0%	0.991
	GALR1 + TLX1	0.79	420	1	49	6	98.6%	98.0%	0.988
	GALR1 + ZNF154	0.91	415	1	49	11	97.4%	98.0%	0.996
	TLX1 + ZNF154	0.54	423	2	48	3	99.3%	96.0%	0.994
	GALR1 + TLX1 + ZNF154	0.80	420	1	49	6	98.6%	98.0%	0.996
KIRC	GALR1	0.40	261	29	131	35	88.2%	81.9%	0.914
	TLX1	0.72	147	5	155	149	49.7%	96.9%	0.753
	ZNF154	0.64	223	6	154	73	75.3%	96.3%	0.898
	GALR1 + TLX1	0.45	240	21	139	56	81.1%	86.9%	0.901
	GALR1 + ZNF154	0.51	250	13	147	46	84.5%	91.9%	0.927
	TLX1 + ZNF154	0.66	228	3	157	68	77.0%	98.1%	0.909
	GALR1 + TLX1 + ZNF154	0.65	244	7	153	52	82.4%	95.6%	0.931

KIRP	GALR1	0.72	100	10	35	56	64.1%	77.8%	0.738
	TLX1	0.72	94	8	37	62	60.3%	82.2%	0.738
	ZNF154	0.77	104	5	40	52	66.7%	88.9%	0.853
	GALR1 + TLX1	0.69	111	11	34	45	71.2%	75.6%	0.765
	GALR1 + ZNF154	0.75	106	6	39	50	67.9%	86.7%	0.855
	TLX1 + ZNF154	0.69	115	7	38	41	73.7%	84.4%	0.860
	GALR1 + TLX1 + ZNF154	0.70	115	7	38	41	73.7%	84.4%	0.859
LIHC	GALR1	0.74	70	3	47	81	46.4%	94.0%	0.635
	TLX1	0.74	103	1	49	48	68.2%	98.0%	0.767
	ZNF154	0.58	131	1	49	20	86.8%	98.0%	0.916
	GALR1 + TLX1	0.72	110	2	48	41	72.8%	96.0%	0.840
	GALR1 + ZNF154	0.54	132	1	49	19	87.4%	98.0%	0.920
	TLX1 + ZNF154	0.41	139	1	49	12	92.1%	98.0%	0.945
	GALR1 + TLX1 + ZNF154	0.43	139	1	49	12	92.1%	98.0%	0.950
LUAD	GALR1	0.81	414	2	30	23	94.7%	93.8%	0.977
	TLX1	0.84	404	1	31	33	92.4%	96.9%	0.971
	ZNF154	0.96	327	0	32	110	74.8%	100.0%	0.912
	GALR1 + TLX1	0.66	429	1	31	8	98.2%	96.9%	0.994
	GALR1 + ZNF154	0.97	412	0	32	25	94.3%	100.0%	0.992
	TLX1 + ZNF154	0.94	419	0	32	18	95.9%	100.0%	0.993
	GALR1 + TLX1 + ZNF154	0.98	425	0	32	12	97.3%	100.0%	0.999
LUSC	GALR1	0.94	292	0	42	67	81.3%	100.0%	0.938
	TLX1	0.81	330	1	41	29	91.9%	97.6%	0.972
	ZNF154	0.87	335	0	42	24	93.3%	100.0%	0.979
	GALR1 + TLX1	0.71	348	1	41	11	96.9%	97.6%	0.990
	GALR1 + ZNF154	0.55	354	0	42	5	98.6%	100.0%	0.993
	TLX1 + ZNF154	0.96	348	0	42	11	96.9%	100.0%	0.995
	GALR1 + TLX1 + ZNF154	0.64	356	0	42	3	99.2%	100.0%	0.996
PAAD	GALR1	0.94	56	0	9	9	86.2%	100.0%	0.967
	TLX1	0.84	55	0	9	10	84.6%	100.0%	0.930
	ZNF154	0.61	63	0	9	2	96.9%	100.0%	0.981
	GALR1 + TLX1	0.79	63	0	9	2	96.9%	100.0%	0.986
	GALR1 + ZNF154	0.58	64	0	9	1	98.5%	100.0%	0.990
	TLX1 + ZNF154	0.68	63	0	9	2	96.9%	100.0%	0.990
	GALR1 + TLX1 + ZNF154	0.58	64	0	9	1	98.5%	100.0%	0.991
PRAD	GALR1	0.81	166	2	47	82	66.9%	95.9%	0.847
	TLX1	0.81	221	5	44	27	89.1%	89.8%	0.935
	ZNF154	0.82	222	6	43	26	89.5%	87.8%	0.918
	GALR1 + TLX1	0.74	228	5	44	20	91.9%	89.8%	0.943

	GALR1 + ZNF154	0.72	229	6	43	19	92.3%	87.8%	0.935
	TLX1 + ZNF154	0.79	223	5	44	25	89.9%	89.8%	0.935
	GALR1 + TLX1 + ZNF154	0.82	224	4	45	24	90.3%	91.8%	0.941
READ	GALR1	0.86	82	0	7	14	85.4%	100.0%	0.894
	TLX1	0.68	93	0	7	3	96.9%	100.0%	0.984
	ZNF154	0.95	75	0	7	21	78.1%	100.0%	0.921
	GALR1 + TLX1	0.61	95	0	7	1	99.0%	100.0%	0.990
	GALR1 + ZNF154	0.93	90	0	7	6	93.8%	100.0%	0.988
	TLX1 + ZNF154	0.50	96	0	7	0	100.0%	100.0%	1.000
	GALR1 + TLX1 + ZNF154	0.50	96	0	7	0	100.0%	100.0%	1.000
STAD	GALR1	0.99	178	0	2	82	68.5%	100.0%	0.821
	TLX1	0.99	209	0	2	51	80.4%	100.0%	0.879
	ZNF154	0.94	255	0	2	5	98.1%	100.0%	0.987
	GALR1 + TLX1	0.99	204	0	2	56	78.5%	100.0%	0.869
	GALR1 + ZNF154	0.92	255	0	2	5	98.1%	100.0%	0.985
	TLX1 + ZNF154	0.78	259	0	2	1	99.6%	100.0%	0.998
	GALR1 + TLX1 + ZNF154	0.50	260	0	2	0	100.0%	100.0%	1.000
UCEC	GALR1	0.95	252	0	46	153	62.2%	100.0%	0.817
	TLX1	0.84	336	0	46	69	83.0%	100.0%	0.903
	ZNF154	0.82	402	0	46	3	99.3%	100.0%	0.997
	GALR1 + TLX1	0.84	348	1	45	57	85.9%	97.8%	0.926
	GALR1 + ZNF154	0.80	401	0	46	4	99.0%	100.0%	0.997
	TLX1 + ZNF154	0.77	401	0	46	4	99.0%	100.0%	0.997
	GALR1 + TLX1 + ZNF154	0.79	401	0	46	4	99.0%	100.0%	0.997

[a] *Tumor types are abbreviated as in Figure 1*
[b] *Probability threshold obtained by applying Youden's Index after logistic regression and ROC curve analysis*
Abbreviations: TP = True Positive, FP = False Positive, TN = True Negative, FN = False Negative, AUC = Area Under the ROC Curve, ROC = Receiver Operating Characteristics
