# Supplementary material for: Evaluating Stacked Methylation Markers for Blood-Based Multicancer Detection
Source: Cancers (Basel). 2023 Oct 1;15(19):4826. doi: 10.3390/cancers15194826 (PMC10571530; doi:10.3390/cancers15194826)
Supplement: Supplementary file 1 [file cancers-15-04826-s001.zip › Table S4.pdf]

Table S4. Logistic regression coefficients from analysis of tumor vs blood samples (10% ctDNA)

			Covariate		
Dilution	Biomarker combination	Intercept	GALR1	TLX1	ZNF154
0.1	G	-1.66	10.83		
0.1	T	-4.12		43.64	
0.1	Z	-2.82			43.81
0.1	G + T	-4.18	0.77	43.03	
0.1	G + Z	-2.95	1.31		42.66
0.1	T + Z	-4.86		32.42	28.17
0.1	G + T + Z	-4.61	-3.88	34.53	30.58
