# Supplementary material for: Evaluating Stacked Methylation Markers for Blood-Based Multicancer Detection
Source: Cancers (Basel). 2023 Oct 1;15(19):4826. doi: 10.3390/cancers15194826 (PMC10571530; doi:10.3390/cancers15194826)
Supplement: Supplementary file 1 [file cancers-15-04826-s001.zip › Table S3.pdf]

Table S3. Measurements of accuracy from 10-fold cross validation logistic regression modelling using tumor vs blood samples

		Cross-validation evaluation			
Biomarker	Dilution level (tumor DNA)	Accuracy	SD (accuracy)	Kappa	SD (kappa)
G	0.01	0.514	0.023	0.028	0.045
T	0.01	0.543	0.022	0.087	0.044
Z	0.01	0.532	0.014	0.065	0.028
GT	0.01	0.532	0.014	0.065	0.028
GZ	0.01	0.532	0.014	0.065	0.028
TZ	0.01	0.532	0.014	0.065	0.028
GTZ	0.01	0.532	0.014	0.065	0.028
G	0.1	0.657	0.014	0.314	0.027
T	0.1	0.792	0.018	0.584	0.035
Z	0.1	0.778	0.016	0.555	0.031
GT	0.1	0.778	0.016	0.555	0.031
GZ	0.1	0.778	0.016	0.555	0.031
TZ	0.1	0.778	0.016	0.555	0.031
GTZ	0.1	0.778	0.016	0.555	0.031
G	0.25	0.772	0.010	0.543	0.021
T	0.25	0.853	0.010	0.706	0.020
Z	0.25	0.855	0.017	0.710	0.033
GT	0.25	0.855	0.017	0.710	0.033
GZ	0.25	0.855	0.017	0.710	0.033
TZ	0.25	0.855	0.017	0.710	0.033
GTZ	0.25	0.855	0.017	0.710	0.033
G	0.5	0.824	0.012	0.648	0.024
T	0.5	0.884	0.011	0.768	0.023
Z	0.5	0.897	0.009	0.794	0.017
GT	0.5	0.897	0.009	0.794	0.017
GZ	0.5	0.897	0.009	0.794	0.017
TZ	0.5	0.897	0.009	0.794	0.017
GTZ	0.5	0.897	0.009	0.794	0.017
G	0.75	0.845	0.019	0.689	0.038
T	0.75	0.895	0.010	0.790	0.021
Z	0.75	0.907	0.010	0.814	0.020
GT	0.75	0.907	0.010	0.814	0.020
GZ	0.75	0.907	0.010	0.814	0.020
TZ	0.75	0.907	0.010	0.814	0.020
GTZ	0.75	0.907	0.010	0.814	0.020
G	0.9	0.852	0.014	0.704	0.027
T	0.9	0.900	0.008	0.799	0.016
Z	0.9	0.911	0.010	0.822	0.020
GT	0.9	0.911	0.010	0.822	0.020
GZ	0.9	0.911	0.010	0.822	0.020
TZ	0.9	0.911	0.010	0.822	0.020
GTZ	0.9	0.911	0.010	0.822	0.020
G	0.99	0.854	0.009	0.709	0.018
T	0.99	0.900	0.012	0.800	0.024
Z	0.99	0.912	0.005	0.825	0.010
GT	0.99	0.912	0.005	0.825	0.010
GZ	0.99	0.912	0.005	0.825	0.010
TZ	0.99	0.912	0.005	0.825	0.010
GTZ	0.99	0.912	0.005	0.825	0.010
G	0.999	0.855	0.013	0.710	0.025
T	0.999	0.900	0.010	0.800	0.020
Z	0.999	0.912	0.010	0.825	0.020
GT	0.999	0.912	0.010	0.825	0.020
GZ	0.999	0.912	0.010	0.825	0.020
TZ	0.999	0.912	0.010	0.825	0.020
GTZ	0.999	0.912	0.010	0.825	0.020
G	1	0.855	0.009	0.710	0.018
T	1	0.900	0.008	0.800	0.017
Z	1	0.913	0.011	0.825	0.022
GT	1	0.913	0.011	0.825	0.022
GZ	1	0.913	0.011	0.825	0.022
TZ	1	0.913	0.011	0.825	0.022
GTZ	1	0.913	0.011	0.825	0.022
