# Supplementary material for: Evaluating Stacked Methylation Markers for Blood-Based Multicancer Detection
Source: Cancers (Basel). 2023 Oct 1;15(19):4826. doi: 10.3390/cancers15194826 (PMC10571530; doi:10.3390/cancers15194826)
Supplement: Supplementary file 1 [file cancers-15-04826-s001.zip › Table S2.pdf]

Table S2. Logistic regression coefficients from analysis of TCGA tumor vs normal tissue samples

			Covariate		
Type	Biomarker combination	Intercept	GALR1	TLX1	ZNF154
BLCA	G	-0.59	6.48		
BLCA	T	0.59		6.77	
BLCA	Z	-0.97			10.72
BLCA	G + T	-1.18	5.52	4.23	
BLCA	G + Z	-1.47	3.26		7.56
BLCA	T + Z	-1.44		4.13	9.20
BLCA	G + T + Z	-1.57	1.79	3.09	7.60
BRCA	G	-0.63	9.20		
BRCA	T	-0.44		8.93	
BRCA	Z	0.22			10.58
BRCA	G + T	-1.62	7.31	6.11	
BRCA	G + Z	-1.64	8.49		7.41
BRCA	T + Z	-1.86		9.28	9.60
BRCA	G + T + Z	-2.56	6.27	6.61	7.44
COAD	G	-2.37	9.60		
COAD	T	-2.50		11.25	
COAD	Z	-3.34			9.42
COAD	G + T	-5.24	8.22	11.13	
COAD	G + Z	-8.39	11.13		10.32
COAD	T + Z	-4.32		11.20	3.57
COAD	G + T + Z	-6.09	8.22	10.75	1.82
HNSC	G	-1.05	8.00		
HNSC	T	-0.90		9.59	
HNSC	Z	-3.39			21.90
HNSC	G + T	-4.87	9.26	11.04	
HNSC	G + Z	-5.75	8.08		19.61
HNSC	T + Z	-4.86		8.50	17.27
HNSC	G + T + Z	-6.32	6.68	6.41	15.30
KIRC	G	-1.18	32.84		
KIRC	T	-1.67		36.81	
KIRC	Z	-5.36			352.81
KIRC	G + T	-2.27	29.50	22.35	
KIRC	G + Z	-5.47	17.83		305.39
KIRC	T + Z	-5.96		17.86	325.45
KIRC	G + T + Z	-5.86	16.83	11.92	289.35
KIRP	G	0.68	10.33		
KIRP	T	-0.51		22.42	
KIRP	Z	-2.29			163.56
KIRP	G + T	-0.66	5.76	20.50	
KIRP	G + Z	-2.30	1.95		159.24
KIRP	T + Z	-2.61		9.81	143.67
KIRP	G + T + Z	-2.63	-1.53	10.14	146.90
LIHC	G	0.32	6.31		
LIHC	T	-0.57		6.82	
LIHC	Z	-1.50			24.02
LIHC	G + T	-1.06	5.68	6.55	
LIHC	G + Z	-1.46	-1.42		25.59
LIHC	T + Z	-2.31		5.90	18.25
LIHC	G + T + Z	-2.22	-4.22	6.49	20.41
LUAD	G	-1.29	23.91		
LUAD	T	-3.32		57.97	
LUAD	Z	-0.26			70.91
LUAD	G + T	-4.72	18.93	40.84	
LUAD	G + Z	-3.58	21.81		67.96
LUAD	T + Z	-6.53		75.25	41.86
LUAD	G + T + Z	-9.42	15.65	76.43	68.11
LUSC	G	-0.72	21.50		
LUSC	T	-3.26		62.42	
LUSC	Z	-4.11			204.98
LUSC	G + T	-4.31	17.65	42.08	
LUSC	G + Z	-4.48	16.78		91.49
LUSC	T + Z	-6.25		64.24	80.20
LUSC	G + T + Z	-6.12	16.61	15.00	110.83
PAAD	G	-1.21	16.78		
PAAD	T	-3.09		48.27	

PAAD	Z	-5.69			273.87
PAAD	G + T	-7.01	15.09	66.82	
PAAD	G + Z	-6.49	6.60		258.10
PAAD	T + Z	-7.43		34.67	226.52
PAAD	G + T + Z	-8.90	6.80	36.39	246.86
PRAD	G	0.22	10.70		
PRAD	T	-1.65		7.98	
PRAD	Z	-1.37			9.42
PRAD	G + T	-1.81	5.05	6.61	
PRAD	G + Z	-1.62	6.10		7.55
PRAD	T + Z	-1.71		6.65	1.88
PRAD	G + T + Z	-1.84	4.92	5.65	1.39
READ	G	0.31	6.98		
READ	T	-2.43		15.46	
READ	Z	-8.27			19.15
READ	G + T	-3.51	6.18	11.12	
READ	G + Z	-20.50	15.61		34.78
READ	T + Z	-768.05		1581.34	917.80
READ	G + T + Z	-288.60	232.14	131.74	385.26
STAD	G	2.16	4.12		
STAD	T	2.30		4.60	
STAD	Z	-0.19			16.88
STAD	G + T	1.74	1.44	3.91	
STAD	G + Z	1.15	-3.04		19.03
STAD	T + Z	9.02		-68.66	153.62
STAD	G + T + Z	3069.70	-4877.41	-10488.81	28880.98
UCEC	G	0.93	5.60		
UCEC	T	-0.21		9.93	
UCEC	Z	-5.76			20.47
UCEC	G + T	-0.51	3.49	8.72	
UCEC	G + Z	-5.59	2.57		18.19
UCEC	T + Z	-5.93		7.40	16.24
UCEC	G + T + Z	-5.91	0.61	7.19	15.97
